# Induction of resilience strategies against biochemical deteriorations prompted by severe cadmium stress in sunflower plant when *Trichoderma* and bacterial inoculation were used as biofertilizers

**DOI:** 10.3389/fpls.2022.1004173

**Published:** 2022-10-20

**Authors:** Amany H. A. Abeed, Rasha E. Mahdy, Dikhnah Alshehri, Inès Hammami, Mamdouh A. Eissa, Arafat Abdel Hamed Abdel Latef, Ghada Abd-Elmonsef Mahmoud

**Affiliations:** ^1^ Botany and Microbiology Department, Faculty of Science, Assiut University, Assiut, Egypt; ^2^ Agronomy Department, Faculty of Agriculture, Assiut University, Assiut, Egypt; ^3^ Department of Biology, Faculty of Science, University of Tabuk, Tabuk, Saudi Arabia; ^4^ Department of Biology, College of Science, Imam Abdulrahman Bin Faisal University, Dammam, Saudi Arabia; ^5^ Department of Soils and Water, Faculty of Agriculture, Assiut University, Assiut, Egypt; ^6^ Department of Botany and Microbiology, Faculty of Science, South Valley University, Qena, Egypt

**Keywords:** adaptability, biofertilizers, growth-promoting bacteria, *Trichoderma harzianum*, *Bacillus subtilis*

## Abstract

**Background:**

Cadmium (Cd) is a highly toxic heavy metal. Its emission is suspected to be further increased due to the dramatic application of ash to agricultural soils and newly reclaimed ones. Thereby, Cd stress encountered by plants will exacerbate. Acute and chronic exposure to Cd can upset plant growth and development and ultimately causes plant death. Microorganisms as agriculturally important biofertilizers have constantly been arising as eco-friendly practices owing to their ability to built-in durability and adaptability mechanisms of plants. However, applying microbes as a biofertilizer agent necessitates the elucidation of the different mechanisms of microbe protection and stabilization of plants against toxic elements in the soil. A greenhouse experiment was performed using *Trichoderma harzianum* and plant growth-promoting (PGP) bacteria (*Azotobacter chroococcum* and *Bacillus subtilis*) individually and integrally to differentiate their potentiality in underpinning various resilience mechanisms versus various Cd levels (0, 50, 100, and 150 mg/kg of soil). Microorganisms were analyzed for Cd tolerance and biosorption capacity, indoleacetic acid production, and phosphate and potassium solubilization *in vitro*. Plant growth parameters, water relations, physiological and biochemical analysis, stress markers and membrane damage traits, and nutritional composition were estimated.

**Results:**

Unequivocal inversion from a state of downregulation to upregulation was distinct under microbial inoculations. Inoculating soil with *T. harzianum* and PGPB markedly enhanced the plant parameters under Cd stress (150 mg/kg) compared with control plants by 4.9% and 13.9%, 5.6% and 11.1%, 55.6% and 5.7%, and 9.1% and 4.6% for plant fresh weight, dry weight, net assimilation rate, and transpiration rate, respectively; by 2.3% and 34.9%, 26.3% and 69.0%, 26.3% and 232.4%, 135.3% and 446.2%, 500% and 95.6%, and 60% and 300% for some metabolites such as starch, amino acids, phenolics, flavonoids, anthocyanin, and proline, respectively; by 134.0% and 604.6% for antioxidants including reduced glutathione; and by 64.8% and 91.2%, 21.9% and 72.7%, and 76.7% and 166.7% for enzymes activity including ascorbate peroxidase, glutathione peroxidase, and phenylalanine ammonia-lyase, respectively. Whereas a hampering effect mediated by PGP bacterial inoculation was registered on levels of superoxide anion, hydroxyl radical, electrolyte leakage, and polyphenol oxidase activity, with a decrease of 0.53%, 14.12%, 2.70%, and 5.70%, respectively, under a highest Cd level (150 mg/kg) compared with control plants. The available soil and plant Cd concentrations were decreased by 11.5% and 47.5%, and 3.8% and 45.0% with *T. harzianum* and PGP bacterial inoculation, respectively, compared with non-inoculated Cd-stressed plants. Whereas, non-significant alternation in antioxidant capacity of sunflower mediated by *T. harzianum* action even with elevated soil Cd concentrations indicates stable oxidative status. The uptake of nutrients, *viz*., K, Ca, Mg, Fe, nitrate, and phosphorus, was interestingly increased (34.0, 4.4, 3.3, 9.2, 30.0, and 1.0 mg/g dry weight, respectively) owing to the synergic inoculation in the presence of 150 mg of Cd/kg.

**Conclusions:**

However, strategies of microbe-induced resilience are largely exclusive and divergent. Biofertilizing potential of *T. harzianum* showed that, owing to its Cd biosorption capability, a resilience strategy was induced *via* reducing Cd bioavailability to be in the range that turned its effect from toxicity to essentiality posing well-known low-dose stimulation phenomena (hormetic effect), whereas using *Azotobacter chroococcum* and *Bacillus subtilis*, owing to their PGP traits, manifested a resilience strategy by neutralizing the potential side effects of Cd toxicity. The synergistic use of fungi and bacteria proved the highest efficiency in imparting sunflower adaptability under Cd stress.

## Introduction

The exceptional change in the environmental conditions due to population explosion, amplified air and soil pollution, ecosystem heavy metal (HM) contamination, depletion of soil quality, and global climate change has considerably impacted the capability of plants to adapt to changing climactic conditions (Dimkpa et al., 2009). These changes make agricultural production systems liable to altering environmental conditions. Such suboptimal environmental conditions can prompt damaging the physiological changes which occur within the plants, called stresses (Raza et al., 2020). Abiotic stresses such as high temperature, metal toxicity, drought, salinity, and nutrient imbalance are those environmental stresses that restrict crop growth and production below the threshold level.

The sunflower (*Helianthus annuus* L.) ranks the fourth most important oilseed crop, following soybean, palm seeds, and canola seeds as a significant source of oil used in various food products worldwide (USDA, FAO, 2008). It is a protein source of great interest for human nutrition, especially due to its sensory, nutritional, and functional properties (Zorzi et al., 2020). In Egypt, the shortage of edible oil represents a huge problem due to the rise in human growth rate with limited oilseed cultivated area at the same time. Therefore, the government is earnestly seeking to increase oilseed crop production, which could be achieved in two ways: horizontal expansion through the cultivation of large newly reclaimed areas or vertical expansion by increasing plant growth rates and production by plant regulators and antioxidants (Hayat et al., 2020). Newly reclaimed soil is likely to explore numerous environmental stress situations, such as nutrient deprivation, low water availability, temperature fluctuations, saline water and soil, high irradiances, and HM contamination.

Available information on the contamination of urban agricultural areas and newly reclaimed ones in Egypt by metals is now widespread (Naggar et al., 2014). According to Lu et al. (2019) and Singh and Steinnes (2020), the maximum allowable concentration for Cd is 0.3 mg/kg, and the toxic soil for plants contains 3–8 mg/kg. However, Naggar et al. (2014) have shown that many agricultural soils in Egypt exceeded the maximum Cd allowable concentrations and reached about 30 mg/kg of soil. This was suspected to be further increased due to the dramatic application of ash to agricultural soils and newly reclaimed ones. Using these agricultural soil types for cultivation will exacerbate the Cd stress encountered by plants; thus, the yield of crops decreases and the quality of field products gets degraded (Abdel Latef, 2013; Hakla et al., 2021; Yaashikaa et al., 2022). Acute and chronic exposure to Cd can induce more damage including severe disturbance and downregulation in the physiological processes of plants, such as increased reactive oxygen and nitrogen species in the cells, which, in turn, act as deleterious molecules mediating an oxidative/nitrosative burst that could cause growth inhibition, accelerated senescence, and mostly cause plant death (Noor et al., 2022). To survive severe Cd stress, the built-in durability and adaptability mechanisms of plants to withstand metal stress conditions are used to improve plant resistance against metal stress, furthermore enhancing the natural role of microorganisms as biofertilizers that will be beneficial in improving soil health and plant productivity (Ortiz and Sansinenea, 2022).

Toward a sustainable agricultural vision and fulfilling HM stress tolerance and better nutritional value, agricultural practitioners are looking increasingly for environmentally friendly inputs such as biofertilizers to manage their crops and cropping systems. Biofertilizers are defined as substances containing living organisms that, when applied to seeds, plant surfaces, or soil, colonize the rhizosphere or the interior of the plant and promote growth by increasing the supply or availability of primary nutrients to the host (Dimkpa et al., 2009). Biofertilizer also refers to the different inoculations of agriculturally beneficial microorganisms (bacteria/fungi) with certain desirable physiological and behavioral characteristics that are utilized for crop nutrition management programs (Zainab et al., 2021). Rhizosphere microorganisms have been used to underpin resilience strategies against abiotic stress in many plants, such as *Solanum nigrum*, *Brassica napus*, tomato, and maize (Dell'Amico et al., 2008; Sheng et al., 2008; Chen et al., 2010; Dourado et al., 2013) mainly achieved by increasing the bioavailability of phosphorus (P) and nitrogen (N) and other soil trace elements essential to plant growth. Moreover, the presence of the symbiotic association also helps to increase plant water uptake; encompasses the adverse effects of phytopathogens; modifies plant growth hormone production; and diminishes the impact of abiotic stresses like drought, salinity, and HM toxicity (Ortiz and Sansinenea, 2022). Their significant role in HM stress management is substantially by eliminating the negative effects of metal on yield quantity and quality (Basu et al., 2017; Ding et al., 2021). They can also accumulate, transform, or detoxify HM.

Consequently, the use of microorganisms that primarily colonizes the rhizosphere/endorhizosphere of plants and hence favors growth directly or indirectly is gaining priority in stress management (Ding et al., 2021). Adaptation and acclimatization of plants for survival under stress conditions due to microorganism inoculations induced physical and chemical changes, wherein the term Induced Systemic Tolerance (IST) has been coined (Basu et al., 2017). This matter catapults them as natural biofertilizers and bioprotection agents (Ortiz and Sansinenea, 2022). However, the underlying strategies of microbe-induced resilience are not well understood, and the modes of action largely remain elusive and microbe species-dependent.

Plant growth-promoting bacteria (PGPB) represent a group of significant microorganisms characterized by plant growth promotion by secreting important compounds such as phytohormones (Gupta et al., 2022) or by increasing nutrient availability of plants (mineralization) (Etesami and Adl, 2020). There is great potential for use of PGPB as biofertilizer agents for a wide variety of crop plants in a wide range of climatic and edaphic conditions. Currently, an array of bacterial inoculates are commercially available for use as biofertilizers or bioprotection against abiotic/biotic stresses (Dasgupta et al., 2021; Kumar et al., 2022). Substantial progress has been exerted in exploring the molecular, physiological, and morphological mechanisms underlying bacterially mediated tolerance to abiotic stresses (Dimkpa et al., 2009; Khan et al., 2021). *Azotobacter and Bacillus* species were common plant growth-promoting (PGP) bacteria (Sheng and Xia, 2006; Abdel-Hakeem et al., 2019). A single PGP bacterium often has multiple uses and modes of action (Rojas-Solis et al., 2020). They impart plant stress tolerance by facilitating the needing resources like fixing N, P, calcium (Ca), potassium (K), and other essential mineral solubilizations and altering plant hormone uptake. Their presence can contribute to the reduction in metal stress on plants when applied to them as single bioinoculants (Wani and Khan, 2010).

The free-living N-fixing bacterium *Azotobacter* secretes biologically active compounds that promote plant growth, such as pantothenic acid, nicotinic acid, B vitamins, biotin, and gibberellin (Patil et al., 2020). *Azotobacter* could promote the growth of plant roots, accelerate the intake of minerals, and compete with other pathogenic microbes (Aasfar et al., 2021). In addition, these are effective phosphate- and K-solubilizing bacteria (Diep and Hieu, 2013); according to Hafez et al. (2016), *Azotobacter* solubilized up to 43% of Egyptian phosphate rock. Singh et al. (2010) stated that *Azotobacter* species can enhance K uptake by plants. Another PGP characteristic of *Azotobacter* is auxin production, which helps the plants for longer roots and increases root hair number and lateral roots, cell division, elongation, and fruit development (Grossmann, 2010; Phillips et al., 2011). In addition, *Bacillus subtilis* has the capacity to solubilize soil P, improve N fixation, and create siderophores that support its growth while inhibiting that of pathogens (Kuan et al., 2016). By promoting the expression of stress-response genes, phytohormones, and stress-related metabolites in their plant hosts, *Bacillus subtilis* improves the tolerance of those hosts to stress (Hashem et al., 2019). According to Kang et al. (2015), *Bacillus* species secrete phosphatases and organic acids that help turn inorganic phosphate into free phosphate by acidifying the environment. *Bacillus* spp. produce chemicals that encourage plant growth, such as indole-3-acetic acid (IAA), gibberellins, cytokinins, and spermidines, and these compounds enhance root and shoot cell division and elongation (Radhakrishnan and Lee, 2016). *Bacillus* species can be introduced to soil that has been contaminated with HMs to lessen the metals’ detrimental effects on plant development and help plants grow by boosting water absorption and lowering electrolyte leaks to lessen Cd stress (Ahmad et al., 2014).

*Trichoderma* has been proven as a potential biofertilization agent to enhance rice plant growth, physiological traits, nutrient uptake, and yield under controlled greenhouse conditions (Doni et al., 2018) when used in the form of a suspension of fungal cells applied to rice seeds or seedlings. They are presently marketed as biopesticides, biofertilizers, growth and yield enhancers, nutrient solubilizers, and organic matter decomposers as well as phytohormones producers, such as IAA, cytokinin, zeatin, and gibberellin. *Trichoderma* species tolerate or detoxify various HMs, especially those microbes from metal-contaminated sites (Zafar et al., 2007). Moreover, it has a high aggregation capability of numerous metals simultaneously, giving it the advantage in use during the bioremediation of soil contaminated with metals (Errasquin and Vazquez, 2003).

Researchers have determined that *Trichoderma* species are important fungi in the reduction of Cd ions. Bazrafshan et al. (2016) and Nongmaithem et al. (2016) confirmed that *Trichoderma* fungus biosorption of Cd (II) ions was spontaneous and endothermic in nature, and *Trichoderma* isolates IBT-I and UBT-18 can tolerant up to 200 ppm. *Trichoderma asperellum* demonstrated a 76.17% effectiveness in removing Cd as recorded by Mohsenzade and Shahrokhi (2014). Cd translocation, tolerance, and absorption in barley have all been linked *to T. harzianum*. It lessens the detrimental effects of Cd pollution and decreases Cd uptake in plants that have received an inoculation of *T. harzianum* (Ghasemkheili et al., 2022). Herliana et al. (2018) found that the highest dry weight of spinach under Cd contamination was recorded in *Trichoderma*-treated plants. According to Marchel et al. (2018), *Trichoderma* inoculation enhanced plant biomass. In addition, Li et al. (2019) stated that plant inoculation with *Trichoderma* improved loofah roots dry weight to 67% comparing with control plants.

*Trichoderma harzianum* represents the most common species of *Trichoderma* that is genetically special, establishes many rhizosphere soils, and could survive in stress conditions for several months (Kubicek et al., 2003). It represents a strong antagonist genus with high soil colonization on the target site and suppresses the other population of pathogenic microorganisms as a biocontrol agent (Hermosa et al., 2012).

This study applied the aspects and mechanisms of HM action of HM-tolerant (HMT) and PGP microbes in ensuring sunflower plant survival and growth in highly Cd-contaminated soils. Using these microbes and studying their interaction with plants in reducing accumulated Cd in plants grown in heavily HM-loaded soil can be the approach for a healthy future. We ascertain the effectiveness of PGPB (*Azotobacter chroococcum* and *Bacillus subtilis*), HMT fungus *Trichoderma harzianum*, and consortium (Mix) treatment on sunflower resurrection, growth, and mitigation of severe Cd stress in agricultural soil. In addition, we assessed the physiological and biochemical sunflower plant responses to the interaction between severe toxic Cd levels and microbe inoculation on accumulated Cd and the performances of a sensitive sunflower cultivar. Thus, knowledge generated from studies on resilience strategies against Cd stress will be very useful in decoding signaling cascades induced by microbial fertilizing agents, resulting in enhanced tolerance and combat versus HM stress.

## Materials and methods

### Plant growth-promoting bacteria

Two bacterial microbes were used in this study. *Azotobacter chroococcum* 14346 and *Bacillus subtilis* 642 were kindly supplemented from Agriculture Research Center, Egypt. The cultures were maintained on nutrient agar (NA) medium (Atlas, 1993) aerobically, stored at 4°C ± 1°C, and subcultured every 4 weeks. The microbes were tested for antagonistic effect on NA plates before use (no antagonistic effect was detected). Prior to the experiments, the bacteria were grown on NA medium at 28°C ± 1°C for 24 h with rotary shaking (200 rpm) until OD660. After that, the bacterial mass was harvested, centrifuged at 6,000×g for 15 min, washed, and suspended in new sterilized water saline with 1 × 10^6^ colony-forming units (CFU)/ml.

### *In vitro* tests for plant growth-promoting bacteria

IAA production was estimated in a mineral broth medium fortified with L-tryptophan (0.2 g/L; Mahmoud and Mostafa, 2017). The sterilized medium was inoculated with bacterial inoculum and incubated for 120 h at 28°C ± 1°C in a rotary shaking incubator at 200 rpm. After that, bacterial cultures were harvested and centrifuged at 6,000×g for 15 min; 1.5 ml of bacterial culture supernatant was mixed with 1 ml of Salkowski reagent pink to red color, which will appear after 30 min according to the IAA concentration (Chrastil, 1976). The developed color was measured using a T60UV split-beam spectrophotometer with wavelength (190–1,100 nm) at 535 nm against free blank and calculated in micrograms per milliliter through the standard curve of pure IAA (1–100 µg/ml). For testing the bacterial ability for phosphate solubilization, Pikovskaya’s agar medium in sterilized petri dishes was inoculated with the microbes and incubated for 120 h at 28°C ± 1°C (Pande et al., 2017). Phosphate solubilization was calculated as the formation of the clear zone (mm) around the bacterial colony, and this diameter was measured every 12 h. For testing the bacterial ability for K solubilization, Aleksandrov’s agar medium in sterilized petri dishes was inoculated with the microbes and incubated for 120 h at 28°C ± 1°C. K solubilization was calculated as the formation of the clear zone (millimeters) around the bacterial colony, and this diameter was measured every 12 h. (Khanghahi et al., 2018). *Azotobacter chroococcum* N-fixing ability was tested on a N-free medium. The microbe was grown on N-free medium plates for 1 week at 28°C ± 1°C incubation temperature; the growth indicated N-fixation capacity (Doroshenko et al., 2007).

### *In vitro* tests for cadmium-resistant fungi

*Trichoderma harzianum* was isolated from HM-contaminated soil on Czapek’s dextrose agar medium supplemented with 50 ppm Cd chloride, identified according to its macroscopic and microscopic properties, preserved aerobically on Czapek’s dextrose agar medium in low temperature at 4°C ± 1°C until use, and subcultured every 3 weeks. For screening the ability of *T. harzianum* to grow on different concentrations of Cd, the agar dilution plate method was used (Ibrahim et al., 2020). Different concentrations of Cd chloride including 0, 50, 100, 150, 200, 250, and 300 ppm were mixed with Czapek’s dextrose agar medium in sterilized petri dishes and left to solidify. *Trichoderma harzianum* 6-mm disc of 3 days growing fungus inoculated on the medium center of the plate and incubated at the moderate temperature at 28°C ± 1°C for a week, and the growth area of the fungus was detected by measuring the colony diameter in millimeters with three replicates. For biosorption activity, *T. harzianum* was grown on Czapek’s dextrose agar medium for 3 days at moderate temperature (28°C ± 1°C); the spores were harvested from the plate surface, mixed with sterilized 0.1% Triton X-100, and diluted to 3 × 10^6^ CFU/ml. Czapek’s dextrose broth medium supplemented with the same Cd concentrations was inoculated with 1% of the fungal spores and incubated at 28°C ± 1°C for 1 week after the fungal broth was used to measure the Cd concentration *via* the Atomic Absorption Spectrophotometer (Buck model 210 VGP, USA) (Mahmoud et al., 2020).

### Experimental design

A greenhouse experiment was performed through a completely randomized design (CRD) with a 6 × 4 factorial plan. Six sunflower plant treatments [with or without PGPB, *T. harzianum*, and consortium (Mix) inoculation] and four Cd soil concentrations with a range of 0–150 mg/kg of soil were used. Four replicates were assessed through each treatment. Each replicate included four plants. Plants exposed to Cd concentrations (50, 100, and 150 mg of Cd/kg of soil) without microbe inoculation underwent severe toxic Cd symptoms ultimately death occurring within 3 days after Cd treatments. Thus, the experiment was completed with plants introduced to interaction between microbe inoculation and the various Cd levels (0, 50, 100, and 150 mg of Cd/kg of soil) only.

### Plant and the microbial treatments

Sunflower (*Helianthus annuus* L) seeds were supplied from the Agronomy Department, Faculty of Agriculture, Assiut University, Egypt. The seeds were sterilized as follows: they were put in 70% ethanol for 2 min and then in 1% NaClO for 10 min, then washed three times with sterilized distilled water, and transferred for germination on sterilized wet filter paper at low temperature (4°C) for 48 h for the synchronized germination; 1-week-old seedlings were further transplanted for the investigation. Microbial inoculums were prepared as s follows: 2-day-old bacterial cells were cultivated in nutrient broth medium, collected by centrifugation at 6,000×g for 15 min, and washed and suspended in new sterilized water saline with 2 × 10^8^ CFU/ml for use. Four-day-old *T. harzianum* cultivated in Czapek’s agar medium was scratched and suspended in sterilized water saline with 3 × 10^6^ CFU/ml inoculum for use. PGPB (*Azotobacter chroococcum* and *Bacillus subtilis*) treatment, *T. harzianum* treatment, and the consortium (Mix) were used for enhancing the growth rate of the sunflower plant under different concentrations of Cd stress (0, 50, 100, and 150 mg of Cd/kg of soil).

### Soil accommodation

Mixed soil samples from 0- to 25-cm depth of sandy loam type were prepared from the surface soil horizon of Assiut University farm; then, the physicochemical properties of the soil were assessed, as shown in **Table 1**. The collected soil was dried in the open air, sieved through 2-mm sieve pores for removing inconstant particles, and autoclaved three times at 121°C for 20 min for 3 consecutive days to remove the native microorganisms. Four Cd concentrations (0, 50, 100, and 150 mg/kg of soil) as Cd dichloride (CdCl_2_) were mixed into the soil as a water solution, and samples were incubated at 20°C for 30 days for Cd distribution and stabilization through the soil layers.

**Table 1:** Physical and chemical characteristics of the used soil.

pH (1:1)	ECdS/m (1:1)	Soluble cations (ppm)				Soluble anions (meq/L)		Particle size distribution (%)		
		Ca^++^	Mg^++^	Na^+^	K^+^	${\text{CO}}_{3}^{-}+{\text{HCO}}_{3}^{-}$	Cl^–^			
7.1	2.25	574	344	186	47	1525	887	Sand 12.4%	Silt 30.9%	Clay 56.7%

### Planting and growth conditions

Pots were then transferred into a greenhouse at 28°C ± 2°C/18°C ± 2°C day/night cycle, 60%–70% relative humidity, and a photoperiod of 14 h. The experimental pots were watered using deionized water once every 3 days to near-field capacity. After 35 days of transplanting, the sunflower plants (42 days old) were harvested by cutting the shoots at the soil surface, and the roots were carefully separated from the soil. The shoots and roots were rinsed with distilled water and wiped with tissue paper.

### Plant growth parameters

Shoot and root length, fresh shoot, and root weight were estimated. For dry shoot and root values, harvested plants were oven-dried at 60°C for 2 days. The leaf area and the net assimilation values were estimated using the adopted methods (Dawood et al., 2019).

### Water relations

Relative water content (RWC) was calculated following the equation adopted by Silveira et al. (2009): RWC = [(FW − DW)/(TW − DW)] **×** 100, where FW is the fresh weight, TW is the turgid weight measured after 24 h of saturation on deionized water at 4°C in the dark, and DW is the dry weight. The transpiration rate was measured as specified by Bozcuk (1975). Leaf stomatal conductance was estimated by adopting the equation recommended by Dawood and Abeed (2020), in which stomatal conductance is expressed as the reverse of the stomatal resistance. The stomatal resistance was measured from the equation displayed by Holmgren et al. (1965) and as modified by Slatyer and Markus (1968). The water use efficiency (WUE) according to Larcher (2003) was determined as follows: WUE (g/kg) = biomass (g/plant)/water use rate (kg/plant).

### Physiological and biochemical analysis

Chlorophyll a, chlorophyll b, and carotenoids were estimated at 663, 644, and 452 nm following the method by Lichtenthaler (1987). Carbon metabolism is evaluated through the detection of glucose and fructose (mg/g DW) as described by Halhoul and Kleinberg (1972), sucrose (mg/g DW) by Van Handel (1968), and starch quantification (mg/g DW) by Fales (1951) and Schlegel (1956). N metabolism was detected by measuring total N, nitrate reductase (NR) activity, amino acids, and proteins following Moore and Stein (1948); Lang (1958); Downs et al. (1993), and Lowry et al. (1951), respectively. Other metabolic molecules were measured as phenolics, flavonoids, anthocyanin, and proline following the methods by Krizek et al. (1993); Kofalvi and Nassuth (1995); Khyade and Vaikos (2009), and Bates et al. (1973), respectively.

### Stress markers and membrane damage traits

Oxidative stress was monitored by determining stress markers such as superoxide anion (μg/g FW, ${\text{O}}_{2}^{\cdot -}$ ), hydroxyl radical (μmol/g FW, ^•^OH), and hydrogen peroxide (μmol/g FW, H_2_O_2_) level in sunflowers leaves, which was quantified as reported by Mukherjee and Choudhuri (1983). Lipid peroxidation was assessed as malondialdehyde (MDA) (μmol/g FW) using the method by Madhava Rao and Sresty (2000). Lipoxygenase (LOX) activity (LOX/EC.1.13.11.1) was assessed at 234 nm according to the method by Minguez-Mosquera et al. (1993). Electrolyte leakage (EL) was estimated by conduct meter (YSI model 35 Yellow Springs, OH, USA) as described by Silveira et al. (2009).

### Non-enzymatic and enzymatic antioxidant capacities

Non-enzymatic antioxidants such as ascorbic acid (ASA) and reduced glutathione (GSH) were assessed following the methods applied by Jagota and Dani (1982) and Ellman (1959), respectively. Phytochelatins (PCs) are determined by the protocols by Ellman (1959) and Nahar et al. (2016). The enzymatic potential of leaves was detected by screening the activities of superoxide dismutase (SOD/EC.1.15.1.1), catalase (CAT/EC 1.11.1.6), ascorbate peroxidase (APX/EC1.11.1.11), glutathione peroxidase (GPX/EC.1.11.1.9), polyphenol oxidase (PPO/EC 1.10.3.1), guaiacol peroxidase (POD/EC 1.11.1.7), phenylalanine ammonia-lyase (PAL/EC 4.3.1.5), and glutathione-S-transferase (GST/EC 2.5.1.18) using the adopted methods by Misra and Fridovich (1972); Nakano and Asada (1981); Kumar and Khan (1983); Aebi (1984); Flohé and Günzler (1984); Tatiana et al. (1999); Sykłowska-Baranek et al. (2012), and Ghelfi et al. (2011), respectively.

### Element composition of the plants

Sodium and K were determined by the flame emission technique (Carl-Zeiss DR LANGE M7D flame photometer) (Havre, 1961). The contents of Ca, Mg, Fe, and Cd were determined with atomic absorption (Shimadzu, model AA-630-02). Nitrate content was quantified by the protocol by Cataldo et al. (1975). P content was estimated spectrophotometrically following the methods by Fogg and Wilkinson (1958).

### Statistical analysis

A CRD was utilized for the pot experiments. Obtained data were expressed as means ± SE. SPSS 10.0 software program was used for performing the statistical analysis. Comparisons between control and treatments were assessed by one-way ANOVA using the least significant difference (LSD) test. Difference from control was counted significant at the probability levels of 0.05 or very significant at the probability levels of 0.01.

## Experimental results

### Plant growth-promoting bacteria

**Figure 1:**
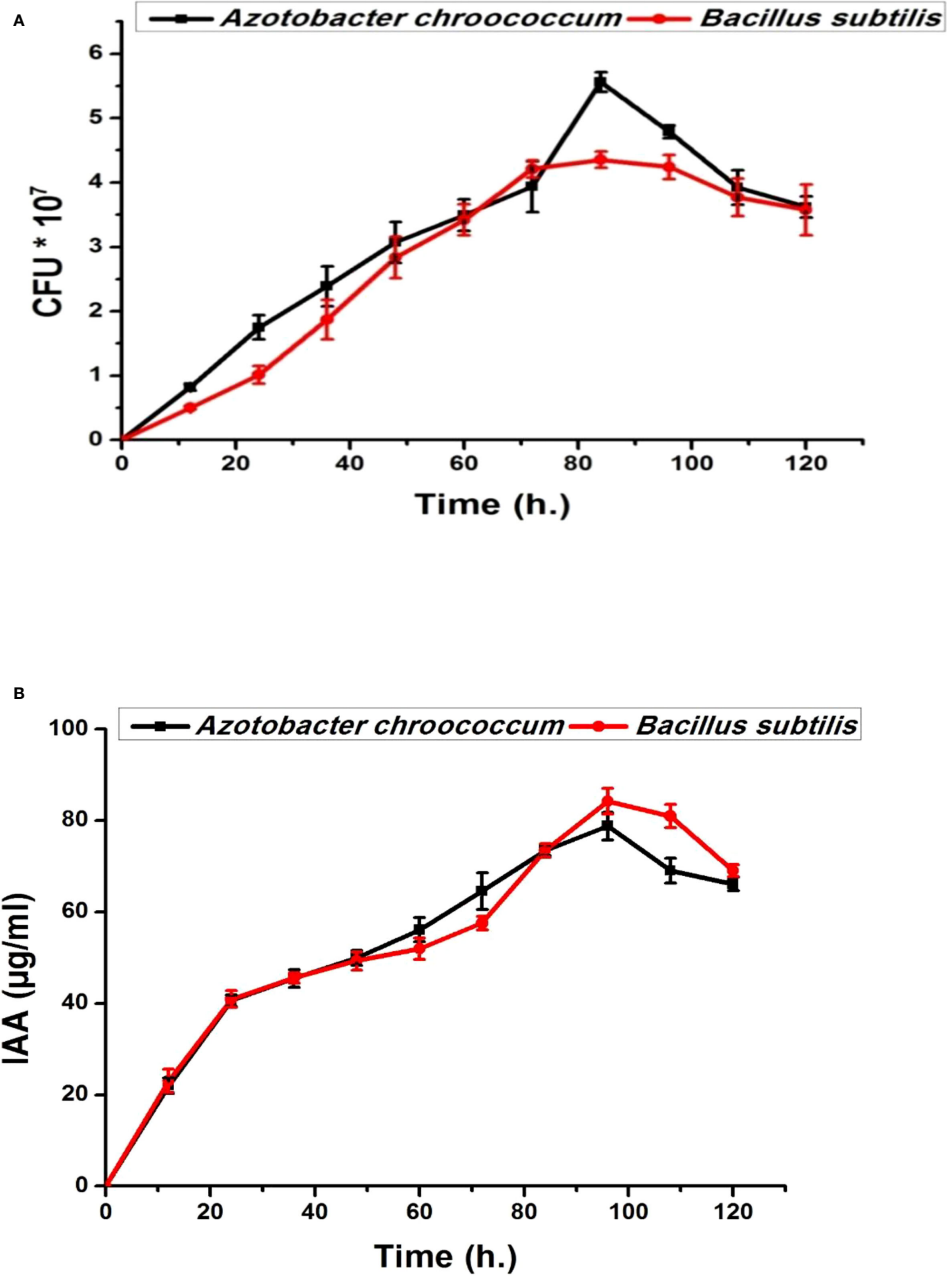
Bacterial growth curve (CFU × 10^7^) **(A)** and indoleacetic acid (IAA) time course production (µg/ml) **(B)** of plant growth-promoting *Azotobacter chroococcum* and *Bacillus subtilis* on nutrient broth medium.

**Figure 2:**
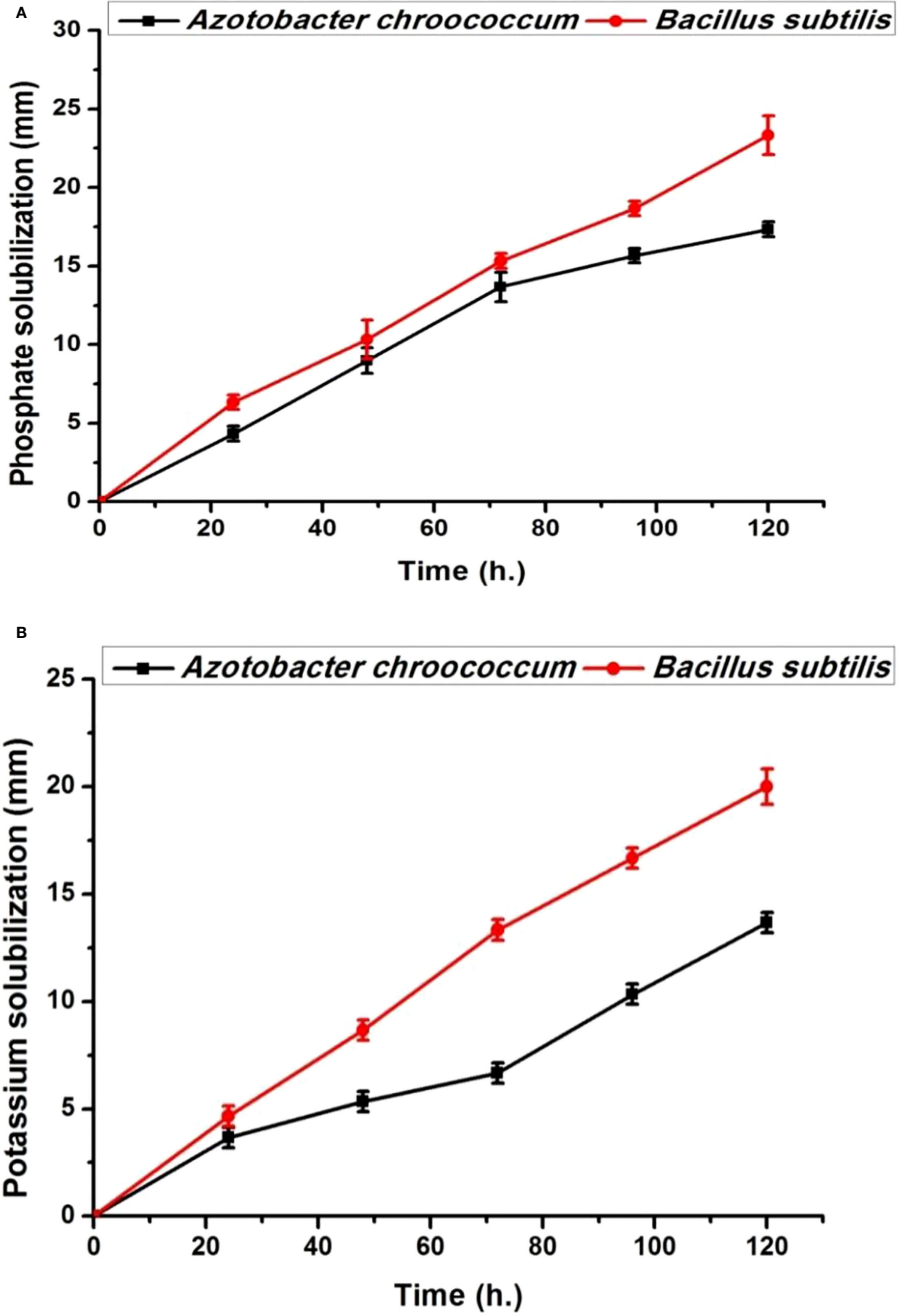
Phosphate solubilization time course as growth diameter (mm) on Pikovskaya’s agar **(A)** and K solubilization on Aleksandrov’s agar **(B)** mediums by the plant growth-promoting (PGP bacteria) *Azotobacter chroococcum* and *Bacillus subtilis*.

Both bacterial strains (*Azotobacter chroococcum* and *Bacillus subtilis*) showed no antagonistic properties with plant growth promotion capabilities through the production of IAA, phosphate, and K solubilization (**Figures 1**, **2**). IAA production starts after 12 h in low quantities for both bacteria and then increased. By increasing the bacterial growth time, IAA production increased in harmony until 96 h and then decreased for both bacteria, giving 78.8 ± 0.98 µg/ml for *A. chroococcum* and 84.27 ± 2.7 µg/ml for *B. subtilis.* However, the maximum CFU for them was 4.79 ± 0.1 × 10^7^ CFU for *A. chroococcum* and 4.36 ± 0.03 ×10^7^ CFU for *B. subtilis* after 96 and 84 h, respectively (**Figures 1A, B**). Both bacterial strains showed high phosphate-solubilizing activities after 24 h of growth and reached their highest values after 120 h with phosphate-solubilizing clear zone of 17.33 ± 0.46 mm for *A. chroococcum* and of 23.3 ± 1.24 mm for *B. subtilis* (**Figure 2A**). K solubilization also takes the same direction as phosphate solubilization, reaching its highest values after 120 h with K solubilizing clear zone of 13.7 ± 0.5 mm for *A. chroococcum* and of 20 ± 0.82 mm for *B. subtilis* (**Figure 2B**). *A. chroococcum* was confirmed as N-fixing bacteria through its growth on a specific N-free medium; both microbes showed no antagonistic properties between each other on NA.

### Cadmium-resistant *Trichoderma*

**Figure 3:**
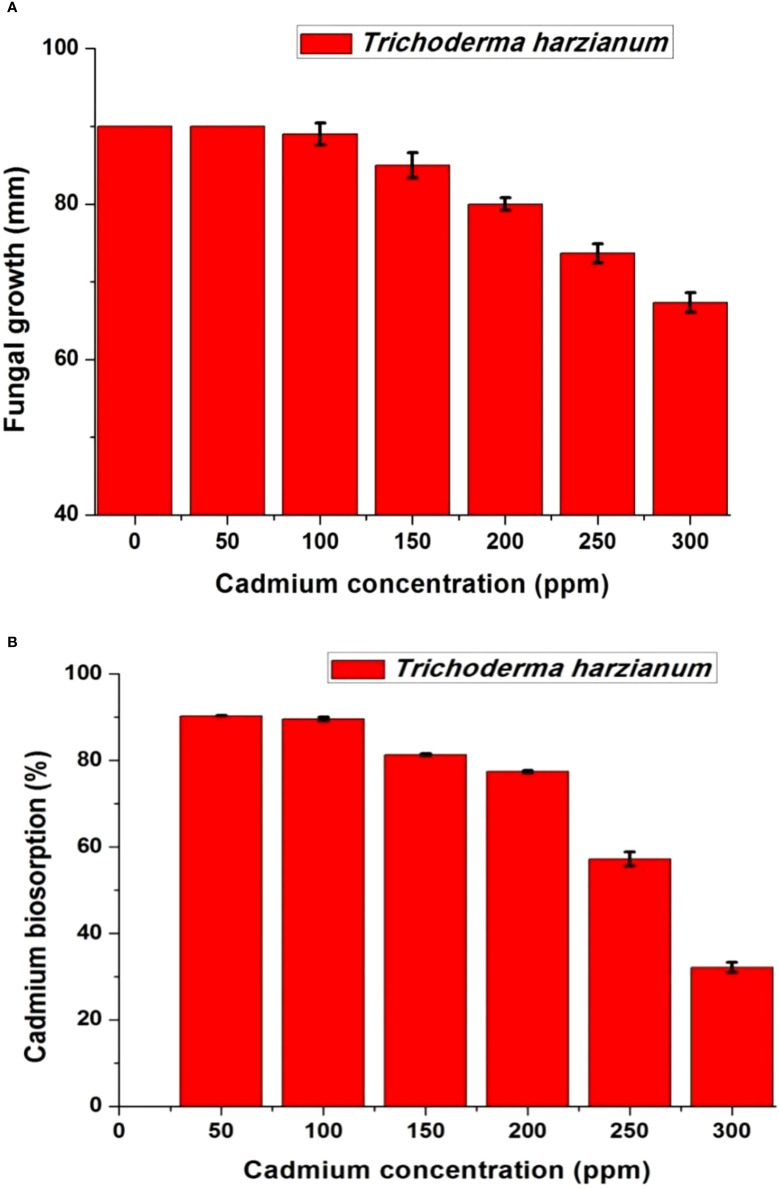
Growth (mm) **(A)** and biosorption percentage (%) **(B)** of cadmium resistance *Trichoderma harzianum* on different concentrations of Czapek’s cadmium medium with cadmium concentrations range from 0 to 300 ppm.

*Trichoderma harzianum* showed the ability to grow on different Cd concentrations in the range of 0–300 ppm as high resistance (**Figures 3A, B**). The growth of *T. harzianum* on Czapek’s agar plates with different Cd concentrations showed that the fungus is slightly affected after 50 ppm and highly affected after 200 ppm. At 50 ppm, the fungus showed complete growth, such as at 0 ppm (90 mm); at 100 ppm, the fungus was slightly affected (89 ± 1.4 mm), and at 200 ppm, it had a growth of 80 ± 0.82 mm. The highest Cd concentration (300 ppm) decreased the growth to 67.3 ± 1.2 mm (**Figure 3A**). The biosorption pattern of *T. harzianum* is indicated in **Figure 3B**; at 50 ppm, the biosorption percentage was 90.32% ± 0.1%, at 100 ppm was 89.54% ± 0.44%, at 150 ppm was 81.35% ± 0.28%, and at 300 ppm was 32.17% ± 1.1%.

### Plant growth and leaf biochemical characteristics derived from cadmium treatments and microbe–soil inoculations interactions

#### Influence of Cd and microbe–soil interaction on morphological and growth attributes of sunflower plant

All Cd concentrations exposure caused fatal damage to plants accompanied by toxic symptoms appearance, including yellowing, chlorosis, stem necrosis, stunting, and wilting. Chlorosis started to appear on leaves after 3 days of Cd exposure and progressed until the end of the treatment, resulting in plant death rather than growth or metabolic retardation, suggesting that plant detoxification processes are insufficient to cope with these lethal concentrations; thus, plants failed to survive the exposure duration. Therefore, the physiological data were obtained from survivor plants in the remaining 13 treatments. Soil microbe inoculations presented a successful approach to reverted plants from distressing to aliveness, thus performing comparably to unstressed plants even at elevated soil Cd levels (up to 150 mg/kg of soil). An initial characterization of the effect of inoculation with microbes was attempted by measuring growth parameters. The resultant plant death due to Cd exposure can effectively be held up by microbe inoculation. Data in **Table 2** showed that the stimulatory effect on plant growth parameters in terms of plant fresh and dry weights (Fwt and Dwt), plant height (PH), and leaf-specific area (LSA) induced by PGPB was more pronounced in non–Cd-stressed plants. Cd stress reduces the beneficial effect of PGPB; however, values of growth parameters registered slight percentages of increase amounted as by 13.9% and 11.1% for plant fresh and dry weights compared with the levels determined for the non-inoculated plants grown on Cd-unpolluted soils. Soil inoculation with *T. harzianum* did not show a stimulatory impact on of non–Cd-stressed plants; however, all detected growth parameters in terms of Fwt, Dwt, PH, and LSA were significantly stimulated by the interaction between *T. harzianum* inoculation and Cd soil existence, particularly in the initial Cd concentration (50 mg/kg of soil). The elevated Cd concentration slightly reduced growth parameters levels compared with those of 50 mg/kg of soil contaminated plants; however, they were still in an acceptable range compared with the control and by percentages of increase amounted as by 4.9% and 5.6% for plant fresh and dry weights. The beneficial effect of soil microbe inoculation was maximized when microbes were in consortium; hence, the highest growth parameters were recorded in the absence or presence of Cd.

**Table 2:** Average values of Fwt, g/plant; Dwt, g/plant; PH, cm; LSA, cm^2^; NAR, µg/cm^2^/d; Sc, mol/m^2^/s; Tr, water loss/leaf area; WUE, g DW/kg H_2_O; RWC, % of sunflower inoculated and non-inoculated with PGP bacteria, *T. harzianum*, and Consortium and affected by different levels of Cd (0, 50, 100, and 150 mg/kg dry soil).

Cd dose (mg/kg)	Inoculation with microbes	Fwt	Dwt	PH	LSA	NAR	Sc	Tr	WUE	RWC
**0**	Non-inoculated	1.44 ± 0.03	0.36 ± 0.001	29.5 ± 1.1	93.33 ± 3.1	0.018 ± 0.002	1.1 ± 0.02	0.022 ± 0.003	3.4 ± 0.4	84 ± 2.1
	PGP bacteria	2.7 ± 0.04d**	0.58 ± 0.001c**	39 ± 1.5c**	110.1 ± 9.1c**	0.032 ± 0.002c**	1.8 ± 0.01c*	0.034 ± 0.002c**	4.5 ± 0.3c**	89 ± 1.9b**
	*T. harzianum*	1.38 ± 0.02a	0.38 ± 0.003a	31.5 ± 2.3a	95.21 ± 5.5a	0.016 ± 0.002a	1.2 ± 0.02a	0.021 ± 0.004a	3.1 ± 0.2a	84 ± 3.1a
	Consortium	2.04 ± 0.03c**	0.46 ± 0.004b*	37 ± 3.3cb**	101.03 ± 11b*	0.044 ± 0.003d**	1.6 ± 0.02b*	0.027 ± 0.005b*	4 ± 0.3b*	90 ± 2.2b**
**50**	Non-inoculated	—	—	—	—	—	—	—	—	—
	PGP bacteria	2.17 ± 0.02c**	0.49 ± 0.001b**	37 ± 1.5cb**	98.67 ± 7.5ab*	0.025 ± 0.001b*	1.6 ± 0.02b*	0.027 ± 0.004b*	4.2 ± 0.2b*	87 ± 5.1ab*
	*T. harzianum*	1.86 ± 0.02b*	0.46 ± 0.003b*	35 ± 1.4b*	100.91 ± 10.5b*	0.030 ± 0.003c**	1.7 ± 0.02bc*	0.031 ± 0.006cb*	4.1 ± 0.3b*	86 ± 3.4ab*
	Consortium	2.50 ± 0.01d**	0.51 ± 0.002cb**	39 ± 2.1c*	107.11 ± 11c**	0.041 ± 0.004d**	2.0 ± 0.03d**	0.034 ± 0.005c**	4.6 ± 0.2c**	90 ± 4.3b**
**100**	Non-inoculated	—	—	—	—	—	—	—	—	—
	PGP bacteria	1.95 ± 0.01b*	0.48 ± 0.001b*	36 ± 1.5b**	95.66 ± 8.7a	0.022 ± 0.002b*	1.4 ± 0.01ab*	0.027 ± 0.003b*	3.9 ± 0.4b*	86 ± 1.7ab*
	*T. harzianum*	1.82 ± 0.01b*	0.42 ± 0.002ab*	31 ± 1.3a	99.03 ± 10.4ab*	0.027 ± 0.003b*	1.5 ± 0.01b*	0.028 ± 0.004b*	3.8 ± 0.3b*	86 ± 2.5ab*
	Consortium	2.24 ± 0.03c**	0.50 ± 0.003cb**	37.5 ± 2.2cb**	100.12 ± 10.3b*	0.037 ± 0.004c**	1.8 ± 0.02c*	0.03 ± 0.005cb*	4.4 ± 0.1cb*	89 ± 2.3b**
**150**	Non-inoculated	—	—	—	—	—	—	—	—	—
	PGP bacteria	1.64 ± 0.02ab	0.40 ± 0.005a	29.5 ± 2.5a	93.11 ± 6.4a	0.019 ± 0.001a	1.1 ± 0.00a	0.023 ± 0.005a	3.2 ± 0.1a	85 ± 3.7a
	*T. harzianum*	1.51 ± 0.03ab	0.38 ± 0.004a	29.7 ± 0.9a	94.56 ± 7.7a	0.028 ± 0.002b*	1.3 ± 0.01ab*	0.024 ± 0.003a	3.3 ± 0.1a	84 ± 4.2a
	Consortium	1.95 ± 0.01b*	0.47 ± 0.002b*	34 ± 3.0b*	97.84 ± 6.6a*	0.025 ± 0.004b*	1.5 ± 0.01b*	0.026 ± 0.003b*	4.2 ± 0.2b*	86 ± 1.1ab*

Fwt,fresh weight; Dwt:dry weight; PH,plant height; LSA,specific leaf area; NAR,net assimilation; Sc,stomatal conductance; Tr,transpiration rate; WUE,water use efficiency rate; RWC,relative water content. Each value represents an average value of three replicates ± SE and averages were compared by LSD at p ≤ 0.05. * and ** denote the difference significantly from the control (0 Cd and non-inoculated) at the probability levels of 0.05 and 0.01,respectively. abc,different letters within columns denote significant differences (P ≤ 0.05) between inoculations with PGP bacteria,T. harzianum, or Consortium in each external Cd level.

#### Influence of Cd and microbe–soil interaction on water relations attributes of sunflower plant

Alleviated Cd toxicity along with soil inoculations can be assessed by analyzing plant–water relation–related factors (**Table 2**). The exhibited trend among the various treatments regarding net assimilation rate (NAR) was increased in food factory units (LSAs). NAR was stimulated by soil inoculated with PGPB in non–Cd-stressed conditions, whereas soil inoculation with *T. harzianum* exhibited stimulatory impact when only Cd existed. Whatever elevated Cd levels were encountered by plants, NAR values were maintained near the levels determined for the non-inoculated plants grown on Cd-unpolluted soils by soil inoculation. The synergistic use of fungi and bacteria in a consortium proved the highest efficiency in enhancing NAR of sunflower plants. Visual toxic appearance including severe wilting resulted in plant death indicated lethal doses encountered by non-inoculated plants. Inoculated soil with PGPB, *T. harzianum*, or in consortium markedly re-stabilized cell water status in terms of RWC (RWC%) that was accompanied by normal transpiration rate and stomatal conductance; thus, efficient water economy in terms of WUE even under elevated Cd levels in the soil indicated effectiveness action of the used microbes under severe Cd stress condition.

#### Influence of Cd and microbe–soil interaction on pigment content and primary and secondary metabolites of sunflower plant

Cd-induced inhibitory effect on photosynthetic machinery *via* depletion of chlorophyll synthesis advocated by apparent chlorosis was significantly ameliorated by the microbe’s inoculation. Microbes’ inoculations reverted chlorophyll contents to those measured for the non-inoculated control even under elevated Cd concentrations (**Table 3**). PGPB-inoculated plants exhibited significant photosynthetic pigment increase under non-Cd stress conditions, whereas the stimulatory effect of *T. harzianum*–inoculated soil was attained by the interaction between *T. harzianum* inoculation and Cd soil existence. Inoculated soil with microbes in consortium posed the highest increase in the level of the photosynthetic pigment in both Cd absence and existence. Carbon metabolism analyzed by quantification of glucose, fructose, and starch was significantly prompted by soil inoculation with PGPB and microbe consortium by increasing the foliar content of glucose, fructose, and starch in both Cd-stressed or non-stressed plants compared with non-inoculated control plants (**Table 3**). Whereas *T. harzianum*–inoculated soil was able to maintain the levels comparable to those determined for non-inoculated control whatever elevated Cd concentrations. Visual Cd toxic symptoms such as necrosis are due to depletion in N uptake in non-inoculated Cd-stressed plants. Plants inoculated with PGPB and consortium registered well-furnished metabolizable N to their body in terms of augmented levels of protein and amino acid as well as enhanced NR activity. However, plants inoculated with *T. harzianum* kept these parameters near the levels of control under Cd-stressed or non-stressed conditions. Low–molecular weight molecules, ***viz***., phenolics, flavonoids, anthocyanin, and proline, were significantly increased by the interaction between Cd stress and microbe inoculation individually or in consortium indicating their role in cellular damage restriction under Cd stress (**Table 3**). The percent of increase accounted as 26.3% and 232.4%, 135.3% and 446.2%, 500% and 95.6%, and 60% and 300%, in the levels of phenolics, flavonoids, anthocyanin, and proline for *T. harzianum* and PGPB, respectively.

**Table 3:** Average values of photosynthetic pigments (Chl a, Chl b, and carotenoids; mg/g FW); sugar metabolism (glucose, sucrose, and starch; mg/g DW); nitrogen metabolism (TN, total nitrogen, mg/g DW; NR, nitrate reductase, μmolNO_2_/g/h; amino acids, mg/g DW; and proteins, mg/g DW) and metabolic molecules (phenolics, mg/g; FW, flavonoids, mg/g FW; anthocyanin, mg/g FW; and proline, mg/g DW) of sunflower inoculated and non-inoculated with PGP bacteria, *T. harzianum*, and Consortium and affected by different levels of Cd (0, 50, 100, and 150 mg/kg dry soil).

Cd dose(mg/kg)	Inoculation with microbes	Photosynthetic pigments			Sugar metabolism			Nitrogen metabolism				Secondary metabolite molecules			
		Chl a	Chl b	Caro	Glucose	Sucrose	Starch	Total nitrogen	NR	Amino acids	Proteins	Phenolics	Flavonoids	Anthocyanin	Proline
**0**	Non-inoculated	0.54 ± 0.02	0.11 ± 0.007	0.66 ± 0.02	17 ± 1.3	51.12 ± 2.5	86 ± 6	30 ± 1.4	60 ± 2.2	22.86 ±	119 ± 7	3.4 ± 0.7	1.3 ± 0.06	0.45 ± 0.003	2.5 ± 0.3
	PGP bacteria	0.89 ± 0.03d**	0.28 ± 0.004d*	1.42 ± 0.1e**	25 ± 1.7b*	78.76 ± 4.5c*	130 ± 9b**	58 ± 1.5d*	110 ± 5.6e**	58.41 ± 2.1d**	134.02 ± 5b*	7.1 ± 0.9d*	2.5 ± 0.03b*	0.55 ± 0.005b*	4 ± 0.3c*
	*T. harzianum*	0.50 ± 0.02a	0.13 ± 0.008a	0.76 ± 0.03b	15 ± 1.1a	55.09 ± 3.3a	89 ± 7.7a	31 ± 0.9a	67.09 ± 1.2a	25.02 ± 1.4a	122 ± 6a	3.6 ± 0.5a	1.7 ± 0.05a	0.43 ± 0.004a	2.1 ± 0.2a
	Consortium	0.71 ± 0.02cb*	0.24 ± 0.009c*	0.95 ± 0.05d*	20 ± 1.8ab*	69.65 ± 2.4b*	150 ± 11.1c**	46 ± 1.1c*	88 ± 1.6c*	42.33 ± 2.1c*	134.89 ± 8b*	4.7 ± 0.4b*	2.4 ± 0.08b*	0.51 ± 0.008b*	3.7 ± 0.2b*
**50**	Non-inoculated	—	—	—	—	—	—	—	—	—	—	—	—	—	—
	PGP bacteria	0.79 ± 0.01c*	0.28 ± 0.01d*	1.22 ± 0.2ed**	23 ± 2.1b*	74.23 ± 3.2c*	122 ± 10ab**	50 ± 0.9d*	86.77 ± 2.2c*	50.70 ± 2.2d*	139 ± 8b*	7.8 ± 0.6ed**	4.9 ± 0.1d*	0.57 ± 0.008b*	6 ± 0.3e**
	*T. harzianum*	0.65 ± 0.01b	0.19 ± 0.006b	0.95 ± 0.05d*	16.03 ± 2.1a	57.12 ± 2.6a	99 ± 6.6a*	40 ± 1.7c*	76.76 ± 1.8b*	33.46 ± 1.5b*	128 ± 5ab	5.6 ± 0.5c*	3.5 ± 0.08c*	0.870.009e**	2.5 ± 0.1a
	Consortium	0.87 ± 0.02d*	0.31 ± 0.008d*	0.99 ± 0.06d*	26 ± 2.2b*	77.33 ± 4.1c**	220 ± 12.6d**	66 ± 2.2e*	95 ± 1.7d*	47.12 ± 1.2c*	136 ± 3b*	5.1 ± 0.7c*	2.5 ± 0.07b*	0.61 ± 0.006c*	4.4 ± 0.1c*
**100**	Non-inoculated	—	—	—	—	—	—	—	—	—	—	—	—	—	—
	PGP bacteria	0.68 ± 0.02b	0.20 ± 0.009b*	0.86 ± 0.05c*	21 ± 1.5b*	70.21 ± 3.1c*	116 ± 10.6a*	44 ± 1.4c*	80 ± 3.2c*	44.09 ± 1.4c*	148 ± 7c**	9.31 ± 0.6f**	5.53 ± 0.07e*	0.67 ± 0.007c*	7.6 ± 0.2f**
	*T. harzianum*	0.55 ± 0.01a	0.16 ± 0.007ab	0.77 ± 0.04b	18 ± 1.1a	63.95 ± 2.6b**	132 ± 10.1b*	34 ± 0.8ab	72 ± 1.7b*	32.77 ± 1.7b*	130 ± 5b*	6.6 ± 0.4d**	3.9 ± 0.06c**	1.9 ± 0.06f**	2.9 ± 0.2a
	Consortium	0.79 ± 0.01c*	0.28 ± 0.008d*	0.89 ± 0.05c*	28 ± 2.2cb*	75.11 ± 2.5c	181 ± 11d*	52 ± 1.7d*	89 ± 1.7c*	48.05 ± 1.5c*	135 ± 3b*	5.5 ± 0.6c*	2.7 ± 0.05b*	0.68 ± 0.005c*	5.3 ± 0.1d*
**150**	Non-inoculated	—	—	—	—	—	—	—	—	—	—	—	—	—	—
	PGP bacteria	0.56 ± 0.005a	0.16 ± 0.009ab	0.73 ± 0.04b	18 ± 2.3ab	61 ± 4.4b*	96 ± 5.5a*	36 ± 0.7b	76 ± 2.1b*	38.64 ± 1.9b*	162 ± 8d**	11.3 ± 0.9g**	7.1 ± 0.2f**	0.88 ± 0.007e**	10.1 ± 0.5g**
	*T. harzianum*	0.51 ± 0.006a	0.12 ± 0.008a	0.65 ± 0.03a	16.76 ± 1.1a	50 ± 2.6a	88 ± 5.7a	31 ± 1.1a	64 ± 1.3a	28.87 ± 1.8a	118 ± 2a	8 ± 0.6e**	4.2 ± 0.05d**	2.7 ± 0.07g**	4 ± 0.2c*
	Consortium	0.74 ± 0.01c*	0.20 ± 0.001b*	0.81 ± 0.05c*	30 ± 3.4c**	66 ± 3.7b*	140 ± 7.5cb**	41 ± 0.8c*	81 ± 3.4c*	45.32 ± 1.5c*	130 ± 3b*	6.7 ± 0.5d**	3.6 ± 0.06c*	0.76 ± 0.006d*	6.1 ± 0.3e**

Each value represents an average value of three replicates ± SE,and averages were compared by LSD at p ≤ 0.05. * and ** denote the difference significantly from the control (0 Cd and non-inoculated) at the probability levels of 0.05 and 0.01,respectively. abc,different letters within columns denote significant differences (P ≤ 0.05) between inoculations with PGP bacteria, T. harzianum ,or Consortium in each external Cd level.

#### Influence of Cd and microbe–soil interaction on oxidative injury and non-enzymatic and enzymatic antioxidants of sunflower plant

The data of reactive oxygen species (ROS) denoted in **Table 4** revealed a decline in superoxide anion and hydroxyl radical by soil microbe inoculation individually or in the consortium, whereas hydrogen peroxide (H_2_O_2_) quietly prompted whatever the dose of Cd applied by PGPB or in the consortium. For example, the decrese is approximately 0.53%, 14.12%, 2.70%, and 5.70% for the levels of superoxide anion ( ${\text{O}}_{2}^{\cdot -}$ ), hydroxyl radical (^•^OH), EL, and PPO activities, respectively, by the action of PGPB. Non-significant alternation in H2O2 level was mediated by *T. harzianum* action even with elevated soil Cd concentrations. The oxidative burst of ROS to cellular membranes was assessed *via* lipid peroxidation in terms of MDA content and LOX, as well as membrane leakage assay of microbe-inoculated Cd-stressed plants, which were found to be similar to the control plants and within the range ensured healthy growth. The upregulation effect of microbe inoculation against Cd stress appeared from low–molecular weight antioxidant metabolism as represented in **Table 4**, where the exacerbation of PCs, ASA, and GSH along with elevated Cd levels in addition to activation of secondary metabolites pathway is illustrated by the overproduction of phenolics, flavonoids, and anthocyanin contents, owing to microbes application. The maintenance of cell oxidative status by enzymatic antioxidants may be due to the increase of superoxide radical dismutation enzyme and SOD, in addition to the prompting of hydrogen peroxide quenching enzymes such as CAT, POD, GPX, APX, and GST, where the maximal activity of these antioxidant enzymes was manifested for plants inoculated with PGPB and in the consortium (**Table 4**). Whereas all antioxidant enzyme activities in leaves of Cd-exposed plants and inoculated with *T. harzianum* exhibited a similar pattern in response to Cd stress and generally showed no significant difference versus the control at 50 and 100 mg/kg, they were slightly stimulated by the highest Cd level (150 mg/kg) in the soil. Plants vastly elicited secondary metabolite-activating enzyme and PAL with microbe inoculation individually or in the consortium by percent increase of 76.7% and 166.7% for *T. harzianum* and PGPB, respectively (**Table 4**). On the other hand, PPO was highly significantly reduced (**Table 4**) for PGPB and in consortium-inoculated plants and non-significantly for *T. harzianum*–inoculated plants relative to non-inoculated control plants.

**Table 4:** Average values of some biochemical indices of sunflower inoculated and non-inoculated with PGP bacteria, *T. harzianum*, and Consortium and affected by different levels of Cd (0, 50, 100, and 150 mg/kg of soil).

Cd dose(mg/kg)	Inoculation with microbes	Membrane integrity traits			Reactive oxygen species			Non- enzymatic antioxidant			Enzymatic antioxidant capacities						
		MDA	LOX	EL	H_2_O_2_	^•^OH	${\text{O}}_{2}^{\cdot -}$	PCs	ASA	GSH	SOD	CAT	POD	APX	GPX	GST	PAL	PPO
**0**	Non-inoculated	53.97 ± 2.3	4.53 ± 0.2	32.91 ± 1.2	170.08 ± 10.1	10.98 ± 0.9	39.44 ± 2.2	20.06 ± 1.1	35 ± 1.1	9.4 ± 0.8	30 ± 0.9	66 ± 1.4	90 ± 4.4	91 ± 3.4	132 ± 10	110 ± 5.9	30 ± 2.1	35 ± 1.9
	PGP bacteria	40.54 ± 2.1*	1.89 ± 0.09**	20.75 ± 1.1*	115.13 ± 9.8*	6.51 ± 0.8*	22.12 ± 2.3*	18.67 ± 1.2	49 ± 2.1*	20 ± 1.7**	44 ± 1.8*	80 ± 3.1*	147 ± 7.7*	114 ± 4.4*	159 ± 10*	100 ± 6.5	55 ± 4.3*	20 ± 1.1*
	*T. harzianum*	51.33 ± 1.4	4.09 ± 0.1	30.23 ± 2.0	165.43 ± 12.2	9.56 ± 0.8	33.06 ± 3.3	23.12 ± 1.4	37 ± 2.4	10 ± 1.3	33 ± 1.1	67 ± 2.5	89 ± 4.3	88 ± 5.6	130 ± 11	108 ± 6.9	28 ± 1.4	32 ± 1.2
	Consortium	43.22 ± 1.1*	2.57 ± 0.2*	22.07 ± 1.2*	120.12 ± 13.1*	5.64 ± 0.4*	21.23 ± 1.3*	20.43 ± 1.1	43 ± 2.4*	22 ± 2.3**	40 ± 1.7*	85 ± 2.6*	100 ± 7.7*	94 ± 6.4	164 ± 9*	111 ± 10	46 ± 2.2*	18 ± 0.9*
**50**	Non-inoculated	—	—	—	—	—	—	—	—	—	—	—	—	—	—	—	—	—
	PGP bacteria	51.76 ± 1.2	2.66 ± 0.1*	29.11 ± 1.1	190 ± 13.1*	7.43 ± 0.5*	32.21 ± 1.4	40.56 ± 2.2*	56.77 ± 2.1*	46 ± 3.4**	48 ± 2.1*	89 ± 4.3*	160 ± 8.5*	133 ± 9.9*	179 ± 10*	170 ± 12.4**	59 ± 3.1*	23 ± 1.3*
	*T. harzianum*	41.21 ± 2.4*	2.01 ± 0.1*	21.43 ± 1.1*	173 ± 12.1	9.32 ± 0.6	37.65 ± 2.1	19.65 ± 1.3	44 ± 1.9*	15 ± 1.8	32 ± 1.7	66 ± 2.1	90 ± 5.5	87 ± 6.4	131 ± 9	100 ± 7.9	35 ± 2.1	25 ± 1.8*
	Consortium	40.43 ± 2.2*	4.04 ± 0.1	25.76 ± 1.3*	120 ± 9.3*	5.43 ± 0.3*	20.32 ± 1.1*	22.21 ± 1.5	49 ± 1.8*	30 ± 2.1**	50 ± 2.4*	87 ± 2.3*	102 ± 6.1*	96 ± 5.7	166 ± 11*	155 ± 9.4*	61 ± 4.3*	16 ± 0.9*
**100**	Non-inoculated	—	—	—	—	—	—	—	—	—	—	—	—	—	—	—	—	—
	PGP bacteria	53.32 ± 1.5	3.87 ± 0.1	30.09 ± 2.2	185 ± 13.1*	9.65 ± 0.8	37.65 ± 2.1	52.33 ± 2.1**	67.98 ± 3.2**	57.98 ± 2.3**	52 ± 2.3*	97 ± 4.3*	161 ± 5.4*	153 ± 10.9*	194 ± 11*	190 ± 11.1**	68 ± 4.1*	29 ± 1.7
	*T. harzianum*	47.65 ± 1.4	4.32 ± 0.8	22.65 ± 1.1	173 ± 11.1	9.98 ± 0.9	25.44 ± 1.5*	20.24 ± 1.1	46 ± 1.8*	18 ± 1.5	33 ± 1.1	67 ± 3.5	88 ± 3.3	80 ± 5.5	133 ± 9	117 ± 10.9	34 ± 3.0	28 ± 1.9
	Consortium	44.56 ± 1.1*	3.87 ± 0.2	27.33 ± 2.1	144 ± 9.7*	6.02 ± 0.7*	22.76 ± 1.1*	30.11 ±	55 ± 2.3*	32 ± 1.7**	54 ± 1.3*	92 ± 3.5*	100 ± 9.9*	99 ± 6.4	157 ± 10*	179 ± 12**	69 ± 4.0*	20 ± 1.1*
**150**	Non-inoculated	—	—	—	—	—	—	—	—	—	—	—	—	—	—	—	—	—
	PGP bacteria	54.45 ± 1.5	4.65 ± 0.9	32.01 ± 1.1	223 ± 17.9*	9.43 ± 0.6	39.23 ± 2.3	58.97 ± 1.9**	87.55 ± 4.3**	66.23 ± 2.4**	61 ± 2.8*	126 ± 5.3*	188 ± 12.2*	174 ± 11*	228 ± 12*	233 ± 14**	80 ± 5.0*	33 ± 2.1
	*T. harzianum*	58.22 ± 1.7	4.08 ± 0.8	34.98 ± 1.7	192 ± 11.4*	10.15 ± 0.8	41.05 ± 2.2	40.12 ± 2.1**	53 ± 2.5*	22 ± 1.2**	39 ± 1.3	78 ± 2.3*	100 ± 8.7*	150 ± 6.8*	161 ± 10*	140 ± 11*	53 ± 4.1*	34 ± 1.5
	Consortium	46.56 ± 1.3	3.77 ± 0.5	29.11 ± 2.1	166 ± 10.9	6.99 ± 0.4*	26.65 ± 1.1*	25.66 ± 1.1	50 ± 1.9*	36 ± 2.1**	59 ± 2.5*	90 ± 3.3*	123 ± 10.1*	110 ± 9.9	170 ± 11*	181 ± 12**	76 ± 5.0*	23 ± 1.2*

MDA,malondialdehyde; LOX,lipoxygenase; EL,electrolyte leakage; H2O2,hydrogen peroxide; OH,hydroxyl radical; O2,superoxide anion; PCs,phytochelatin; GSH,reduced glutathione; ASA,ascorbic acid; CAT,catalase; SOD,superoxide dismutase; POD,guaiacol peroxidase; APX,ascorbate peroxidase; GPX,glutathione peroxide; GST,glutathione-S-transferase; PAL,phenylalanine ammonia-lyase; PPO,polyphenol oxidase. Each value represents an average value of three replicates ± SE,and averages were compared by LSD at p ≤ 0.05. * and ** denote the difference significantly from the control (0 Cd and non-inoculated) at the probability levels of 0.05 and 0.01,respectively. abc,different letters within columns denote significant differences (P ≤ 0.05) between inoculations with PGP bacteria,T. harzianum,or Consortium in each external Cd levels.

#### Influence of Cd and microbe–soil interaction on leaf nutrient content of sunflower plant

Cd in the nutrient medium was deleterious to nutrient uptake, ***viz***., K, Ca, Mg, Fe, nitrate, and P; however, data illustrated in **Table 5** showed that microbe inoculation, even in the presence of Cd, increased all of these parameters to be more than or very close to those of the control.

**Table 5:** Average values of Leaf Nutrient content (K, Mg, Ca, Fe, nitrate, and phosphorus) of sunflower inoculated and non-inoculated with PGP bacteria, *T. harzianum*, and Consortium and affected by different levels of Cd (0, 50, 100, and 150 mg/kg dry soil).

Cd doses (mg/kg)	Inoculation with microbes	Leaf Nutrient content (mg/g DW)					
		K	Ca	Mg	Fe	Nitrate	Phosphorus
0	Non-inoculated	22 ± 1.9	4.0 ± 0.2	3.3 ± 0.1	9 ± 0.6	29 ± 2.5	0.81 ± 0.02
	PGP bacteria	45 ± 3.3d**	7.8 ± 0.5e**	4 ± 0.1b*	12 ± 0.8g*	34 ± 2.4f*	1.6 ± 0.08b**
	*T. harzianum*	33 ± 2.6b*	5.1 ± 0.4c	2.9 ± 0.1a	6 ± 0.5a*	28 ± 1.8a	0.95 ± 0.03a*
	Consortium	39 ± 1.5c**	5.9 ± 0.3c*	3.8 ± 0.3b	9.3 ± 0.5d	33 ± 3.5e*	2 ± 0.1c**
50	Non-inoculated	—	—	—	—	—	—
	PGP bacteria	41 ± 1.1cd**	5 ± 0.1c	4 ± 0.2b*	11 ± 0.8f*	32 ± 1.9d*	1.5 ± 0.08b**
	*T. harzianum*	34 ± 2.1b*	6.5 ± 0.2d**	3.5 ± 0.1ab	7.5 ± 0.6b*	33 ± 1.3e*	1.2 ± 0.06ab*
	Consortium	43± 1.7d**	5.5 ± 0.2c*	4.4 ± 0.2b*	10 ± 0.9e	32 ± 2.4d*	1.9 ± 0.09cb**
100	Non-inoculated	—	—	—	—	—	—
	PGP bacteria	39 ± 1.7c**	4 ± 0.1b	5 ± 0.3c**	9 ± 0.5d	29 ± 1.5a	1 ± 0.08a*
	*T. harzianum*	37 ± 1.3c**	6.8 ± 0.1d**	3.1 ± 0.1a	10 ± 0.6e	31 ± 1.7c*	0.92 ± 0.05a*
	Consortium	40 ± 2.3cd**	5 ± 0.1c	4 ± 0.1b*	9 ± 0.4d	30 ± 1.7b	2.2 ± 0.1c**
150	Non-inoculated	—	—	—	—	—	—
	PGP bacteria	40 ± 2.6cd**	3 ± 0.2a	3 ± 0.2a	8.2 ± 0.5c	31 ± 1.1c*	0.82 ± 0.04a
	*T. harzianum*	44 ± 2.7d**	7.2 ± 0.1 e**	5.3 ± 0.3 c**	12 ± 0.6 g*	34 ± 1.4 f*	1.2 ± 0.06 a*
	Consortium	34 ± 2.1b*	4.4 ± 0.2b	3.3 ± 0.2a	9.2 ± 0.5 d	30 ± 1.0 b	1. 0 ± 0.06 a*

Each value represents an average value of three replicates ± SE,and averages were compared by LSD at p ≤ 0.05. * and ** denote the difference significantly from the control (0 Cd and non-inoculated) at the probability levels of 0.05 and 0.01,respectively. abc,different letters within columns denote significant differences (P ≤ 0.05) between inoculations PGP bacteria, T. harzianum,or Consortium in each external Cd levels. * and ** denote the difference significantly from the control (0 Cd and non-inoculated) at the probability levels of 0.05 and 0.01,respectively. abc,different letters within columns indicate significant differences (P ≤ 0.05) between inoculations PGP bacteria, T. harzianum,or Consortium in each external Cd levels.

#### Influence of Cd and microbe–soil interaction on Cd concentrations in soil and sunflower plant

Data depicted in **Table 6** showed that, by increasing the Cd concentration, the available soil and plant Cd increase. However, microbial fortification decreases the Cd soil and plant availability compared with control samples (free from applied microorganisms) by 11.5% and 47.5%, and 3.8% and 45.0% with *T. harzianum* and PGP bacterial inoculation, respectively, compared with non-inoculated Cd-stressed plants. The microbial consortium was the most efficient treatment that decreases Cd availability in soil.

**Table 6:** Cadmium concentrations in soil and sunflower plants inoculated and non-inoculated with PGP bacteria, *T. harzianum*, and Consortium after being affected by different doses of Cd (0, 50, 100, and 150 mg/kg of soil).

Cd dose (mg/kg)	Inoculation with microbes	Available Cd concentration (mg/kg)	
		Soil	Plant
0	Non-inoculated	0.05 ± 0.001a	0.42 ± 0.01c
	PGP bacteria	0.06 ± 0.001a	0.32 ± 0.01b
	*T. harzianum*	0.03 ± 0.000a	0.24 ± 0.02a
	Consortium	0.04 ± 0.001a	0.20 ± 0.02a
50	Non-inoculated	4.5 ± 0.06c	20 ± 0.21d
	PGP bacteria	3.6 ± 0.08b	16 ± 0.22c
	*T. harzianum*	3.0 ± 0.07a	10 ± 0.12b
	Consortium	3.2 ± 0.08a	6 ± 0.16a
100	Non-inoculated	10.2 ± 0.21d	27 ± 0.62c
	PGP bacteria	8.4 ± 0.11b	18 ± 0.22b
	*T. harzianum*	7.4 ± 0.23a	14 ± 0.24a
	Consortium	9 ± 0.22c	13 ± 0.33a
150	Non-inoculated	13.3 ± 0.12c	40 ± 0.67c
	PGP bacteria	12.5 ± 0.13bc	22 ± 0.87b
	*T. harzianum*	11.5 ± 0.11b	21 ± 0.55b
	Consortium	10.2 ± 0.11a	13 ± 0.64a

Each value represents an average value of three replicates ± SE,and averages were compared by LSD at p ≤ 0.05. abc,different letters within columns indicate significant differences (P ≤ 0.05) between inoculations PGP bacteria, T. harzianum ,or Consortium in each external Cd level.

## Discussion

The potential of PGPB (*Azotobacter chroococcum* and *Bacillus subtilis*) and *Trichoderma harzianum* was investigated individually or synergistically for the first time regarding their capability to impart tolerance in sunflower plant for withstanding and survival to severe Cd dose up to 150 mg of Cd/kg of soil. Excessive concentrations of HMs upset various biochemical pathways in plants, such as inhibition of chlorophyll synthesis, photosynthesis, respiration, transpiration rates, N metabolism, uptake of nutrient elements, cell elongation, changes in photoassimilates translocation, hormone balance *via* a diminution of the endogenous level of growth-promoting hormone auxin, which happened due to boosted activity of the auxin-degrading enzyme, and alteration of water relations, which further enhance the metal-induced growth reduction; consequently, plant death was the result (Kumar and Trivedi, 2016; Eissa and Abeed, 2019; Zhu et al., 2020). Evidence indicates the fatal stress imposed by the applied doses upon sunflower plant resulted in plant death rather than limited growth or even reduced cell performance. Pretreatment of soil with microbes lessen the applied dose toxicity and enables sunflower plant to adjust its metabolism to overcome the fatal stress, manifesting evident plant liveliness, and prompted safely leaf characteristics (e.g., aliveness, greenness, and moisture profile) and also neutralized all growth and physiological parameters, leading to values comparable with those of the control. A trend of *Azotobacter chroococcum–* and *Bacillus subtilis*–inoculated soil submitted higher improving impact on sunflower plant than *Trichoderma harzianum–*inoculated soil under Cd-stressed or non-stressed conditions, whereas consortium treatment assures the highest improving impact along with elevated Cd doses. However, the imparted tolerance mechanism mediated by the two different inoculations (PGPB and HMT fungus) was distinct from each other according to their PGR traits and Cd biosorption capacities, whereas the consortium exhibited dual benefits.

The current findings revealed that the tolerance mechanism mediated by *Azotobacter chroococcum* and *Bacillus subtilis* was due to PGR traits. PGR traits regarding growth hormone (IAA) production and their high ability to solubilize P and K, displaying growth enrichment and intensified 1ry and 2ry metabolite production that was pronounced in the presence and absence of Cd. The high production level of IAA was actively maintained during Cd exposure as a responded hormone in *Azotobacter chroococcum* and *Bacillus subtilis.* This accomplished a continuous supply of IAA to sunflower along with elevated Cd doses that promoted photosynthetic pigments and plant biomass acquisition in terms of dry/fresh weights, PH, LSA, and NAR in sunflower plants under Cd stress. Consistent with our findings, *Azotobacter* and *Bacillus* species proved their ability to enhance plant growth by producing significant compounds such as IAA (Ahmad et al., 2008). Khan et al. (2009) and Kumar et al. (2001) revealed that *Azotobacter chroococcum* can solubilize phosphate and produce phytohormones as kinetin, gibberellin, and IAA P solubilization. Zaidi et al. (2006) stated that *Bacillus subtilis* produces IAA in addition to its ability to mineralize the unavailable phosphate in the soil. Most ***Azotobacter*** species can convert the state of atmospheric N to plant-usable (ammonia) through biological N fixation (Kim and Rees, 1994). A marked increase in plant germination, length, and weight was achieved by applying phosphate-solubilizing and N-fixing *Azotobacter* species (Widawati, 2018). Soil application with *Azotobacter chroococcum* increased cotton seed yield by 21% and PH by 5% (Anjum et al., 2007), in addition to protecting *Brassica juncea* from HM toxicity by increasing plant growth (Wu et al., 2006). Fortification of soil by PGP *Bacillus* species significantly boosted chickpea plant growth, chlorophyll, and yield and reduced metal uptake (Wani and Khan, 2010). *Bacillus subtilis* increased tomato biomass by 31%, okra by 36%, and spinach by 83% (Adesemoye et al., 2008).

On the other hand, *Trichoderma harzianum* has the unequivocal capacity to bind Cd (efficient Cd biosorption) counted for about 81.35% ± 0.28% at the highest Cd soil existence (150 mg of Cd/kg of soil; **Figure 3B**). Metal bioavailability is an important factor for metal uptake in plants. Reducing Cd bioavailability percentage in the rooting portion *via* the retention of HMs by fungal mycelia involves adsorption to cell walls or immobilizing them by insoluble metal oxalate formation or chelation on melanin-like polymers (Khan et al., 2017). Consequently, a minor amount of exposed Cd will be encountered by the plant, thus minimizing metal root uptake and low translocation level to the shoots. This hypothesis was corroborated by Kapoor and Bhatnagar (2007), who demonstrated that fungal mycelia had a high metal sorption capacity. Mycelia attenuate the toxic effect of metals *via* retaining them in the fungal structure with the subsequent restriction of metal transfer to the plant (Joner et al., 2000). Biosorption includes surface adsorption, ion chelation, ion exchange, and micro-precipitation (Lim et al., 2008). Fungal cell surfaces contain functional groups like carboxyl, amino, hydroxyl, and phosphate, which play a critical role in Cd ion uptake (Lim et al., 2008). Mohsenzade and Shahrokhi (2014)found that *T. harzianum* could adsorb Cd from 1 to 100 mg/L. *T. harzianum* could grow on different Cd concentrations (0–300 ppm) with huge tolerance, especially in high concentrations; in addition, it can absorb high quantities of Cd to reach 90% removal efficiency. Consistent with our results, Lima et al. (2011) demonstrated the *T. harzianum* potentiality of Cd removal, which increased by increasing the Cd concentration from 1 to 3 mM. The tolerance of *Trichoderma* to HMs can be explained by biosorption or bioaccumulation processes (Mahmoud, 2021) and metal binding to microbial biomass (Nair et al., 2008). Hence, *T. harzianum* application alleviated the toxic effects of Cd by accumulating and/or immobilizing most Cd in their biomass extracellularly, resulting in developing trace soil Cd concentration that remains free and available for plant roots after *T. harzianum* action witnessed in our study by the reduced total amount of Cd accumulated per plant (**Table 6**). Therefore, the bioprotective effect of *T. harzianum* on sunflower growth was due to a reduced amount of Cd uptake owing to biosorption property rather than the PGP traits; hence, the promoter effect of *T. harzianum* was sounded only in the presence of Cd in soil medium. Here, the positive effect on plant growth could be due to the low bioavailable Cd concentration, posing well-known low-dose stimulation phenomena (hormetic effect). Documented promoted growth was shown because of 10 mg of Cd/kg of soil (Jia et al., 2012; Jia et al., 2015) and 125 mg of Cd/kg of soil (Liu et al., 2012) in *Lonicera japonica*. This significant stimulating effect on growth parameters regarding PH, fresh and dry weights, and the specific leaf area after *T. harzianum* action was realized up to 150 mg of Cd/kg of soil (**Table 2**), indicating the ability of *T. harzianum* to tolerate Cd and acting actively even at lethal doses for the plant (Nongmaithem et al., 2016; Hoseinzadeh et al., 2017). A positive effect on plant growth at low Cd concentration has also been registered in plants, such as barley (Wu et al., 2003), miscanthus (Arduini et al., 2004), soybean (Sobkowiak and Deckert, 2003), and rice (Aina et al., 2007).

In the presence of Cd, chlorosis symptom appearance indicating chlorophyll degradation and plant death was registered in our study. Moreover, necrosis might have been caused by ROS production and membrane dysfunction, which eventually results in programmed cell death. Moreover, the enhanced chlorophyll content and function in inoculated Cd-stressed plants witnessed by high photoassimilates content and subsequent Cd stress recovery indicated that Cd stress tolerance herein may be attained through chlorophyll restoration against elevated Cd supplementation, which could be a promising mechanism to trigger sunflower tolerance against severe Cd stress mediated by microbe inoculation, and the main sensitivity criterion of the current tested sunflower cultivar was photosynthetic depletion under Cd stress and enhanced chlorophyll function and composition linked with a reversed plant from distressed to aliveness. This might be attributed to adequate Mg uptake due to the link between chlorophyll content and Mg uptake as an important part of the chlorophyll molecule (Sheng et al., 2008). Thus, for enhanced Chl a and b contents in PGPB- and *T. harzianum*–inoculated plants, the subsequent recovery of Cd-stressed plants could be accounted for by the effect of these microbes on Mg uptake (Shahabivand et al., 2017). Jia et al. (2015) reported that the stimulating effect of Cd (10 mg/kg) on plant biomass could be ascribed to the increment recorded in photosynthetic carbon assimilation, and the low doses of Cd induced some beneficial effects on the photosynthetic system and increased pigment contents, demonstrating that low doses of Cd induced some beneficial effects on the photosynthetic apparatus. Moreover, Zhou and Qiu (2005) manifested the main explanation of the increase in chlorophyll content at a low Cd level due to a Cd-induced increase in Fe uptake. The other mechanism that may be mediated by *T. harzianum* inoculation as a biofertilizer to confer metal stress tolerance herein is *via* an increase in nutrient uptake under Cd stress. On the other hand, Bashri and Prasad (2015) reported that IAA liberated by PGPB could prompt the downregulation of pigment degradation, causing substantial preservation of pigment content (Abeed et al., 2020; Li et al., 2021).

Stomatal conductance and transpiration rate displayed adequate levels and comparable values of control due to *T. harzianum* that has high Cd-binding capacity, resulting in low available soil Cd. Similar results were submitted for corn plants exposed to 25 mM Cd (Chaneva et al., 2010). Furthermore, the adequate NAR along with Cd treatments submitted by *T. harzianum* inoculation could be ascribed to a high net photosynthetic rate. Ying et al. (2010) cited that increased photosynthesis could be due to elevated Rubisco content at low levels of Cd treatment, evidencing the positive effect of Cd by *T. harzianum* interaction. Moreover, data from water relations indicated that the concentration of HMs in inoculated soil was efficiently lowered to reach a level that causes no osmotic disturbances in plants. Thus, adequate soil–water relation leads to improved water uptake and economic use of water evidenced by adequate water status of the cell regarding RWC and high value of WUE. In different way and apart from *T. harzianum*, the IAAproducing microbe, enhanced root systems, including root hairs, are the most common phenotypic phenomena observed related to the secretion of phytohormones by PGP microbes. Thus, IAA enhanced the capability of plants to exploit the water from the soil in the highest concentration of soil Cd. Consequently, enhanced root growth and performance lead to improved water uptake and economic water use evidenced by adequate water status of the cell regarding RWC and high value of WUE.

Carbon and N resource use and photoassimilate production can be assessed by adopting some carbon and N metabolites and their related enzymes. In the current study, microbe’s soil inoculations manifested adequate carbon and N metabolism that was reflected in starch and protein contents. As sucrose is the famous exported form of organic carbon transport from the photosynthetic source to sink organ in turn, high accumulation of starch indicated high efficiency of using carbon resources and photoassimilate production in favor of plant architecture, and this process is crucial for survival and healthiness as submitted in current investigated plants (Koch, 2004). NR is one of the coordinative enzymes that regulated the level of N in plants. The activity of this enzyme is downregulated by the presence of Cd (Gouia et al., 2000) as evident herein by minimized inducible rate (**Table 3**), resulting in an alternation in protein metabolism that negatively affected plant architecture witnessed by stunted plants (**Table 2**). Inoculated with PGPB, *T. harzianum*, individually and in the consortium, can significantly increase N metabolism as evidenced by the increased total N in shoots reflected in the total N yield of inoculated plants because all fixed N is incorporated into the plants, resulting in high protein content as N is an integral part of proteins in Cd-stressed and non-stressed plant. In well-adapted plants, enhanced primary metabolism goes along with secondary metabolism augmentation. In the current study, the activation of the secondary metabolite pathway, as proved by the exacerbation of phenolics, flavonoids, and anthocyanin, was a significant feature of tolerance mechanism and adaptation in sunflower plant attained by microbe inoculations, hence providing powerful free-radical quenching antioxidants and larger antioxidant defense pools to restraint ROS toxicity, thereby no membrane dysfunction. The upregulation of the main biosynthetic pathways of these antioxidants was joined with the enhanced activity of secondary metabolites, regulating enzymes and PAL, and this was vastly sounded by microbe inoculation rather than non-inoculated control plants.

The correlation of proline in Cd stress tolerance is a major biochemical adaptation, membrane stabilization, and ROS scavenging involved in the chelation of Cd (Ahmad et al., 2016). The mediation inoculation with microbes remarkably increased proline content in sunflowers. In the current study, the exacerbation of proline was accompanied by the increment of amino acids and soluble protein production. This obvious proline accumulation was certainly to be profitable, not due to a harmful impact. Thus, it could be concluded that proline accumulation was a plant response associated with conferring metal tolerance, not a reaction to high Cd exposure, confirming the protective effect of microbe inoculation in neutralizing toxic ROS, therefore contributing to better growth under Cd exposure. Similar to our results, Hui et al. (2015) also recorded an increase in proline accumulation in *Nicotiana tabacum* due to *P. indica* inoculation under Cd stress conditions.

One vital strategy of avoidance of Cd-induced oxidative stress is *via* Cd complexation either by glutathione or PCs. They dropped the free availability of Cd in the cytosol, causing significant tolerance against Cd toxicity (Yamazaki et al., 2018). This was efficiently mediated by IAA-producing microbes. IAA enhanced the level of PCs in the cytosol as reported by Khare et al. (2022). Moreover, the improved nutritional status that was registered by improving Fe uptake after bacterial inoculation indicates possible action of PGPB in Cd deposition out of important metabolic processes (in vacuoles), which may clarify the reduction of Cd phytotoxic impact despite the increased Cd accumulation; hence, the total amount of Cd accumulated per plant in PGPB-inoculated plants was significantly higher than that in *T. harzianum*–inoculated plants (**Table 6**); however, no phytotoxic appearance was recorded. The Fe-dependent transporters that are responsible for transporting PC-Cd-S complex to vacuoles (Hall and Williams, 2003) are adequately available herein owing to improving Fe uptake due to PGP bacteria application. Furthermore, the abundance in PC biosynthesis can be explained by abundant glutathione as registered in the current study because glutathione is the substrate for PC biosynthesis (Yamazaki et al., 2018).

The abatement of both ROS and their toxic byproducts (oxidized proteins and lipid hydroperoxides) is a prerequisite for the survival of plants in the existence of toxic metals. The produced IAA by PGPB was demonstrated to alleviate H_2_O_2_ and ${\text{O}}_{2}^{\cdot -}$ under Cd stress; hence, decreased lipid peroxidation (protection of cell membrane) was observed (Bashri and Prasad, 2015). This was postulated by elevated antioxidant enzyme activities joined with abatement of H_2_O_2_, ^•^OH, and ${\text{O}}_{2}^{\cdot -}$ contents, indicating an IAA-induced ameliorating effect inducing the expression of stress-responsive genes and enhanced the antioxidant levels (Khare et al., 2022). The strong activation of GST in the case of PGPB-inoculated plants advocates the crucial role of PGPB in the Cd-induced stress response, in which IAA might play an important signaling role in participating in the activation of GST. Previously, it has been shown that the expression of many GSTs is strongly activated by IAA (Bočová et al., 2013). The increment of proteins and free amino acids due to PGPB-inoculated soil may be attributed to the activation of stress proteins that include several antioxidant enzymes (Lamhamdi et al., 2011) witnessed by elevated activity of ROS-metabolizing enzymes under either Cd-stressed or non-stressed plants; thus, stimulating the defense system machinery helped the plant to orchestrate itself from damage up to threshold; in addition, eliciting the expression of low–molecular weight proteins comprised the metal ion homeostasis that is assumed to shoulder role in their detoxification, ***viz***., ASA, GSH (acting as a substrate of APX and GPX, respectively), and tocopherol (Patel et al., 2012). PPO is related to stress conditions and involves the cell wall cross-linking and lignification process resulting in a reduction in cell wall extensibility, which restricts cell growth, revealing exhausted plant tissues (Bruce and West, 1989; Abeed and Salama, 2022). PPO oxidatively breaks up phenolic compounds included in the synthesis of quinines and ROS; thus, the promotion of PPO activity exacerbates oxidative stress. In the current investigation, fortunately, the data for the PPO activity were divergent from the other antioxidant enzymes. PPO, an oxidizer of phenolic compounds  (Queiroz et al., 2008; Abeed et al., 2021), is not induced by IAA-producing microbe inoculation but rather dropped in its content compared with non-inoculated control plants; thus, the upregulation of PPO due to soil inoculation under Cd stress diminishes PPO activity in sunflower plant that offered to promote resistance to abiotic stress (Sánchez-Rodríguez et al., 2011).

On the other hand, regarding *T. harzianum* action, much more sensitive parameters, such as biochemical parameters, should be analyzed to evaluate the stimulatory effect induced by the developed low Cd concentration by soil inoculation with *Trichoderma harzianum*. As no quantity or quality of toxic symptoms was noticed, what was the mechanism of the corresponding alterations in redox status in the inoculated plants? The stress causative agents (free radical components) were progressively decreased in the available soil Cd concentration by *T. harzianum* action (**Table 4**), indicating that no oxidative stress was imposed by the remaining concentrations of Cd in the soil (counted by about 14% reduction from non-inoculated control plant, **Figure 3B**) due to *T. harzianum* action that advocated by low MDA content and LOX activity that reflected on membrane stability and integrity evidenced by low electric leakage value. Similar findings were detected by Lin et al. (2007) and Maksymiec et al. (2007). Consequently, well-functional membranes with adequate integrity and tight controlled permeability can be maintained, thus efficiently reducing water loss and providing high turgidity and firmness and optimum water status for metabolic activities that are evidenced by values of WUE and RWC comparable with that of control (**Table 2**). Furthermore, no changes in ROS quenching enzymes activities, *viz*., SOD, CAT, POD, APX, GPX, and GST activities under the developed low Cd concentrations, displayed similar responses to Cd treatments, probably due to their co-regulation, indicating no excess accumulation of ROS in sunflower plants inoculated by *T. harzianum* because their activity is mediated by generated ROS level. Somashekaraiah et al. (1992) documented that the SOD activity mediated by superoxide level exhibited a slight drop or no change linked with no excess accumulation of superoxide anion in mung bean seedlings under low levels of Cd stress. Wu et al. (2003) also found a slight decrease in antioxidant capacities accompanied by a decrement in barley lipid peroxidation products with a low-level Cd dose. The abatement of H_2_O_2_ and ^•^OH and stabilization of ${\text{O}}_{2}^{\cdot -}$ production in the Cd stress plant inoculated with *T. harzianum* reflected that the Cd levels remained in the soil owing to *T. harzianum* action are in an acceptable extent that harmfully impact plants. This may also suggest that the stimulatory effects of low concentrations of Cd on the growth of sunflower plants may be joined with a limited degree of free radical accumulation and restricted oxidative stress (Lin et al., 2007). However, when organisms are subjected to low Cd concentrations, their intrinsic GSH might be rapidly consumed because of a high cellular prerequisite for SH compounds to resist stress by prompting PC synthesis (Yamazaki et al., 2018), which may explain the increasing PCs content in our study owing to *T. harzianum* action. On the basis of the results of the current study regarding oxidative status corresponding to the available Cd concentration (mg/kg) in soil and plant, we propose that the toxic critical value of soil Cd in inducing oxidative stress in sunflower plant is 10.2 and 13 mg/kg for soil and plant (**Table 6**), respectively. This was efficiently achieved by soil supplementation with microbes. Similar results were registered for wheat seedlings by Lin et al. (2007).

The enhanced nutritional status of sunflower plant due to *T. harzianum* inoculation can be explained by the observation of Liu et al. (2011), who demonstrated that there is a synergistic interaction in accumulation and translocation between Cd and Fe, Zn, Mn, and Mg uptake in *L. japonica* plant. They have been improved at low Cd concentrations. The competition for the same uptake systems between Cd and other divalent ions required for plant development is minimized in low Cd soil existence (Liu et al., 2011). However, the nourishment of the nutrient content of plant leaves due to PGP inoculation could be ascribed to the IAA generated by PGPB that modifies membrane permeability (Mir et al., 2022), which, in turn, might have facilitated the uptake of N, P, Mg, Zn, and Fe, resulting in the exacerbation of their levels, even in the plants exposed to Cd stress.

## Conclusions

Applying microbes as a biofertilizer agent necessitates the elucidation of the different mechanisms of microbe protection and stabilization of plants against toxic elements in the soil that may be varied according to their PGR traits and/or Cd-binding capacities. Overall, our findings indicate that the two microbes have differentially established and maintained healthy physiological and biochemical properties of plants cultivated in severe Cd doses (divergent imparted upregulation mechanisms displayed by the two microbes used on sunflower plant adaptation). The high ability of PGPB to produce IAA, which was actively maintained during Cd exposure as a responded hormone, accomplished a continuous supply of IAA to sunflower along with elevated Cd doses (extended for 5 days giving values of 78.8 µg/ml for *Azotobacter chroococcum* and of 84.27 µg/ml for *Bacillus subtilis*) that subsequently promoted photosynthetic pigments and plant biomass acquisition in terms of dry/fresh weights, PH, LSA, and NAR as well as improved the other assessed physiological traits in sunflower plants under Cd stress. Thus, the resilience strategy mediated by PGPB was *via* recovering the potential side effects of Cd toxicity. Whereas, the highly Cd-tolerant *Trichoderma harzianum* with high Cd biosorption capacity (counted as 81.35% at the highest Cd soil existence) induced a resilience strategy *via* reducing Cd bioavailability to be in the range that turned its effect from toxicity to essentiality (the available soil and plant Cd concentrations were decreased by 11.5% and 47.5%, respectively), posing well-known low-dose stimulation phenomena (hormetic effect). However, the consortium exhibited dual benefits, achieving the highest efficiency in the resurrection of sunflower under severe Cd levels.

## Data availability statement

The original contributions presented in the study are included in the article/supplementary material, further inquiries can be directed to the corresponding authors.

## Author contributions

AA and GM: conceived the experiments; performed the experiments; analyzed and interpreted the data; contributed to reagents, materials, analysis tools, or data; and wrote the paper. AA, GM, ME, and RM: materials, experimental analysis, and experimental design. ME, RM, DA, IH, and AL: experimental analysis, writing, revising, and editing. All authors contributed to the article and approved the submitted version.

## Acknowledgments

The authors would like to acknowledge the Department of Botany and Microbiology, Faculty of Science, Assiut University; and Soil and Water Department, Faculty of Agriculture, Assiut University, for supporting this work.

## Conflict of interest

The authors declare that the research was conducted in the absence of any commercial or financial relationships that could be construed as a potential conflict of interest.

## Publisher’s note

All claims expressed in this article are solely those of the authors and do not necessarily represent those of their affiliated organizations, or those of the publisher, the editors and the reviewers. Any product that may be evaluated in this article, or claim that may be made by its manufacturer, is not guaranteed or endorsed by the publisher.

## Abbreviations

PGP, plant growth promoting; HMT, heavy metal tolerant; IAA, indole-3-acetic acid; Fwt, fresh weight; Dwt, dry weight; PH, plant height; LSA, specific leaf area; NAR, net assimilation rate; Sc, stomatal conductance; Tr, transpiration rate; WUE, water use efficiency; RWC, relative water content; TN, total nitrogen content; NR, nitrate reductase; MDA, malondialdehyde; LOX, lipoxygenase; EL, electrolyte leakage; ROS, reactive oxygen species; H_2_O_2_, hydrogen peroxide; ^•^OH, hydroxyl radical; ${\text{O}}_{2}^{\cdot -}$ , superoxide anion; PCs, phytochelatin; GSH, reduced glutathione; ASA, ascorbic acid; CAT, catalase; SOD, superoxide dismutase; POD, guaiacol peroxidase; APX, ascorbate peroxidase; GPX, glutathione peroxide; GST, glutathione-***S***-transferase; PAL, phenylalanine ammonia-lyase; PPO, polyphenol oxidase.

## References

[B1] Aasfar A. Bargaz A. Yaakoubi K. Hilali A. Bennis I. Zeroual Y.. (2021). Nitrogen fixing azotobacter species as potential soil biological enhancers for crop nutrition and yield stability. Front. Microbiol. 12. doi: 10.3389/fmicb.2021.628379 PMC7947814 33717018

[B2] Abdel-Hakeem S. S. Mahmoud G. A.-E. Abdel-Hafeez H. H. (2019). Evaluation and microanalysis of parasitic and bacterial agents of Egyptian fresh sushi, salmo salar. Microscopy Microanal. 25, 1498–1508. doi: 10.1017/s143192761901506x 31718724

[B3] Abdel Latef A. A. (2013). Growth and some physiological activities of pepper (Capsicum annuum l.) in response to cadmium stress and mycorrhizal symbiosis. J. Agric. Sci. Technol. 15, 1437–1448. Available at: http://jast.modares.ac.ir/article-23-11530-en.html

[B4] Abeed A. H. A. Ali M. Ali E. F. Majrashi A. Eissa M. A. (2021). Induction of catharanthus roseus secondary metabolites when calotropis procera was used as bio-stimulant. Plants 10, 1623. doi: 10.3390/plants10081623 34451668 PMC8398584

[B5] Abeed A. H. A. Eissa M. A. Abdel-Wahab D. A. (2020). Effect of exogenously applied jasmonic acid and kinetin on drought tolerance of wheat cultivars based on morpho-physiological evaluation. J. Soil Sci. Plant Nutr. 21, 131–144. doi: 10.1007/s42729-020-00348-1

[B6] Abeed A. H. A. Salama F. M. (2022). Attenuating effect of an extract of cd-hyperaccumulator solanum nigrum on the growth and physio-chemical changes of datura innoxia under cd stress. J. Soil Sci. Plant Nutr 22. doi: 10.1007/s42729-022-00966-x

[B7] Adesemoye A. O. Obini M. Ugoji E. O. (2008). Comparison of plant growth-promotion with pseudomonas aeruginosa and bacillus subtilis in three vegetables. Braz. J. Microbiol. 39, 423–426. doi: 10.1590/s1517-83822008000300003 24031240 PMC3768429

[B8] Aebi H. (1984). Catalase *in vitro* . Method Enzymol. 105, 121–126. doi: 10.1016/S0076-6879(84)05016-3 6727660

[B9] Ahmad P. Abdel Latef A. A. Abd_Allah E. F. Hashem A. Sarwat M. Anjum N. A.. (2016). Calcium and potassium supplementation enhanced growth, osmolyte secondary metabolite production, and enzymatic antioxidant machinery in cadmium-exposed chickpea (Cicer arietinum l.). Front. Plant Sci. 7. doi: 10.3389/fpls.2016.00513 PMC4847423 27200003

[B10] Ahmad F. Ahmad I. Khan M. S. (2008). Screening of free-living rhizospheric bacteria for their multiple plant growth promoting activities. Microbiol. Res. 163, 173–181. doi: 10.1016/j.micres.2006.04.001 16735107

[B11] Ahmad I. Akhtar M. J. Zahir Z. A. Naveed M. Mitter B. Sessitsch A. (2014). Cadmium-tolerant bacteria induce metal stress tolerance in cereals.Environ. Sci. pollut. Res. 21, 11054–11065. doi: 10.1007/s11356-014-3010-9 24849374

[B12] Aina R. Labra M. Fumagalli P. Vannini C. Marsoni M. Cucchi U.. (2007). Thiol-peptide level and proteomic changes in response to cadmium toxicity in oryza sativa l. roots. Environ. Exp. botany. 59, 381–392. doi: 10.1016/j.envexpbot.2006.04.010

[B13] Anjum M. A. Sajjad M. R. Akhtar N. Qureshi M. A. Iqbal A. Rehman J. A.. (2007). Response of cotton to plant growth promoting rhizobacteria (PGPR) inoculation under different levels of nitrogen. J. Agric. Res. 45, 135–143. doi: 10.1007/978-3-319-13401-7_1

[B14] Arduini I. Masoni A. Mariotti M. Ercoli L. (2004). Low cadmium application increase miscanthus growth and cadmium translocation. Environ. Exp. Botany. 52, 89–100. doi: 10.1016/j.envexpbot.2004.01.001

[B15] Atlas R. M. (1993). Handbook of microbiological media (Boca Raton, FL: CRC Press). doi: 10.1201/ebk1439804063

[B16] Bashri G. Prasad S. M. (2015). Indole acetic acid modulates changes in growth, chlorophyll a fluorescence and antioxidant potential of trigonella foenum-graecum l. grown under cadmium stress. Acta physiologiae plantarum. 37, 49. doi: 10.1007/s11738-014-1745-z

[B17] Basu S. Rabara R. Negi S. (2017). Towards a better greener future-an alternative strategy using biofertilizers. I: Plant growth promoting bacteria. Plant Gene. 12, 43–49. doi: 10.1016/j.plgene.2017.07.004

[B18] Bates L. Waldren R. Teare I. (1973). Rapid determination of free proline for water-stress studies. Plant Soil. 39, 205–207. doi: 10.1007/BF00018060

[B19] Bazrafshan E. Zarei A. A. Mostafapour F. K. (2016). Biosorption of cadmium from aqueous solutions by trichoderma fungus: kinetic, thermodynamic, and equilibrium study. Desalination Water Treat 57, 14598–14608. doi: 10.1080/19443994.2015.1065764

[B20] Bočová B. Huttová J. Mistrík I. Tamás L. (2013). Auxin signalling is involved in cadmium-induced glutathione-s-transferase activity in barley root. Acta physiologiae plantarum. 35, 2685–2690. doi: 10.1007/s11738-013-1300-3

[B21] Bozcuk S. (1975). “Effect of sodium chloride upon growth and transpiration in statice sp. and pisum sativum l,” in Proceedings of the 3rd MPP meetings, vol. 75. (Izmir, Turkey: Ege university, Izmir, Turkey), 37–42.

[B22] Bruce R. J. West C. A. (1989). Elicitation of lignin biosynthesis and isoperoxidase activity by pectic fragments in suspension cultures of castor bean. Plant Physiol. 91, 889–897. doi: 10.1104/pp.91.3.889 16667153 PMC1062092

[B23] Cataldo D. A. Maroon M. Schrader L. E. Youngs V. L. (1975). Rapid colorimetric determination of nitrate in plant tissue by nitration of salicylic acid. Commun. Soil Sci. Plant analysis. 6, 71–80. doi: 10.1080/00103627509366547

[B24] Chaneva G. Parvanova P. Tzvetkova N. Uzunova A. (2010). Photosynthetic response of maize plants against cadmium and paraquat impact. Water Air Soil pollut. 208, 287–293. doi: 10.1007/s11270-009-0166-x

[B25] Chen L. Luo S. Xiao X. Guo H. Chen J. Wan Y.. (2010). Application of plant growth-promoting endophytes (PGPE) isolated from solanum nigrum l. for phytoextraction of cd-polluted soils. Appl. Soil Ecol. 46, 383–389. doi: 10.1016/j.apsoil.2010.10.003

[B26] Chrastil J. (1976). Colorimetric estimation of indole-3-acetic acid. Anal. Biochem. 72, 134–138. doi: 10.1016/0003-2697(76)90514-5 942043

[B27] Dasgupta D. Kumar K. Miglani R. Mishra R. Panda A. K. Bisht S. S. (2021). Microbial biofertilizers: Recent trends and future outlook. Recent Adv. Microbial Biotechnol., 1–26. doi: 10.1016/B978-0-12-822098-6.00001-X

[B28] Dawood M. F. A. Abeed A. H. A. (2020). Spermine-priming restrained water relations and biochemical deteriorations prompted by water deficit on two soybean cultivars. Heliyon 6, e04038. doi: 10.1016/j.heliyon.2020.e04038 32509989 PMC7264753

[B29] Dawood M. F. A. Abeed A. H. A. Aldaby E. E. S. (2019). Titanium dioxide nanoparticles model growth kinetic traits of some wheat cultivars under different water regimes. Plant Physiol. Rep. 24, 129–140. doi: 10.1007/s40502-019-0437-5

[B30] Dell'Amico E. Cavalca L. Andreoni V. (2008). Improvement of brassica napus growth under cadmium stress by cadmium-resistant rhizobacteria. Soil Biol. Biochem. 40, 74–84. doi: 10.1016/j.soilbio.2007.06.024

[B31] Diep C. N. Hieu T. N. (2013). Phosphate and potassium solubilizing bacteria from weathered materials of denatured rock mountain, ha tien, kiên giang province Vietnam. Am. J. Life Sci. 1, 88–92. doi: 10.11648/j.ajls.20130103.12

[B32] Dimkpa C. Weinand T. Asch F. (2009). Plant–rhizobacteria interactions alleviate abiotic stress conditions. Plant Cell Env. 32, 1682–1694. doi: 10.1111/j.1365-3040.2009.02028.x 19671096

[B33] Ding Z. Ali E. F. Almaroai Y. A. Eissa M. A. Abeed A. H. (2021). Effect of potassium solubilizing bacteria and humic acid on faba bean (Vicia faba l.) plants grown on sandy loam soils. J. Soil Sci. Plant Nutr. 21, 791–800. doi: 10.1007/s42729-020-00401-z

[B34] Doni F. Zain C. R. C. M. Isahak A. Fathurrahman F. Anhar A. Mohamad W. N. A. W.. (2018). A simple, efficient, and farmer-friendly trichoderma-based biofertilizer evaluated with the SRI rice management system. Organ. Agricul. 8, 207–223. doi: 10.1007/s13165-017-0185-7

[B35] Doroshenko E. V. Boulygina E. S. Spiridonova E. M. Tourova T. P. Kravchenko I. K. (2007). Isolation and characterization of nitrogen-fixing bacteria of the genus azospirillum from the soil of a sphagnum peat bog. Microbiology 76, 93–101. doi: 10.1134/S0026261707010134 17410881

[B36] Dourado M. N. Martins P. F. Quecine M. C. Piotto F. A. Souza L. A. Franco M. R.. (2013). Burkholderia sp. SCMS54 reduces cadmium toxicity and promotes growth in tomato. Ann. Appl. Biol. 163, 494–507. doi: 10.1111/aab.12066

[B37] Downs M. R. Nadelhoffer K. Melillo J. J. Aber J. (1993). Foliar and fine root nitrate reductase activity in seedlings of four forest tree species in relation to nitrogen availability. Trees 7, 233–236. doi: 10.1007/bf00202079

[B38] Eissa M. A. Abeed A. H. (2019). Growth and biochemical changes in quail bush (Atriplex lentiformis (Torr.) s. wats) under cd stress. Environ. Sci. pollut. Res. 26, 628–635. doi: 10.1007/s11356-018-3627-1 30411292

[B39] Ellman G. L. (1959). Tissue sulfhydryl groups. Arch. Biochem. Biophys. 82, 70–77. doi: 10.1016/0003-9861(59)90090-6 13650640

[B40] Errasquin E. L. Vazquez C. (2003). Tolerance and uptake of heavy metals by trichoderma atroviride isolated from sludge. Chemosphere 50, 137–143. doi: 10.1016/s0045-6535(02)00485-x 12656239

[B41] Etesami H. Adl S. M. (2020). “Plant growth-promoting rhizobacteria (PGPR) and their action mechanisms in availability of nutrients to plants,” in Phyto-microbiome in stress regulation. environmental and microbial biotechnology. Eds. Kumar M. Kumar V. Prasad R. (Singapore: Springer). doi: 10.1007/978-981-15-2576-6_9

[B42] Fales F. (1951). The assimilation and degradation of carbohydrates by yeast cells. J. Biol. Chem. 193, 113–124. doi: 10.1016/s0021-9258(19)52433-4 14907695

[B43] Flohé L. Günzler W. A. (1984). “Methods in enzymology,” in Assays of glutathione peroxidase. Ed. Packer L. (New York: Academic Press), 114–121. 10.1016/s0076-6879(84)05015-1 6727659

[B44] Fogg D. N. Wilkinson N. T. (1958). The colorimetric determination of phosphorus. Analyst. 83, 406. doi: 10.1039/an9588300406

[B45] Ghasemkheili T. F. Ekelund F. Johansen J. L. Pirdashti H. Shiade S. R.G. Fathi A.. (2022). Ameliorative effects of trichoderma harzianum and rhizosphere soil microbes on cadmium biosorption of barley (Hordeum vulgare l.) in cd-polluted soil. J. Soil Sci. Plant Nutr. 22, 527–539. doi: 10.1007/s42729-021-00666-y

[B46] Ghelfi A. Gaziola S. A. Cia M. C. Chabregas S. M. Falco M. C. Kuser-Falcão P. R.. (2011). Cloning, expression, molecular modelling and docking analysis of glutathione transferase from saccharum officinarum. Ann. Appl. Biol. 159, 267–280. doi: 10.1111/j.1744-7348.2011.00491.x

[B47] Gouia H. Ghorbal M. H. Meyer C. (2000). Effects of cadmium on activity of nitrate reductase and on other enzymes of the nitrate assimilation pathway in bean. Plant Physiol. Biochem. 38, 629–638. doi: 10.1016/s0981-9428(00)00775-0

[B48] Grossmann K. (2010). Auxin herbicides: current status of mechanism and mode of action. Pest Manage. Sci. 66, 113–120. doi: 10.1002/ps.1860 19823992

[B49] Gupta A. Mishra R. Rai S. Bano A. Pathak N. Fujita M.. (2022). Mechanistic insights of plant growth promoting bacteria mediated drought and salt stress tolerance in plants for sustainable agriculture. Int. J. Mol. Sci. 23, 3741. doi: 10.3390/ijms23073741 35409104 PMC8998651

[B50] Hafez M. Elbarbary T. A. Ibrahim I. Abdel-Fatah Y. (2016). Azotobacter vinelandii evaluation and optimization of Abu tartur Egyptian phosphate ore dissolution. Saudi J. Pathol. Microbiol. 1, 80–93. doi: 10.21276/sjpm.2016.1.3.2

[B51] Hakla H. R. Sharma S. Urfan M. Yadav N. S. Rajput P. Kotwal D.. (2021). Gibberellins target shoot-root growth, morpho-physiological and molecular pathways to induce cadmium tolerance in vigna radiata l. Agronomy 11, 896. doi: 10.3390/agronomy11050896

[B52] Halhoul M. N. Kleinberg I. (1972). Differential determination of glucose and fructose, and glucose- and fructose-yielding substances with anthrone. Anal. Biochem. 50, 337–343. doi: 10.1016/0003-2697(72)90042-5 4646057

[B53] Hall J. L. Williams L. E. (2003). Transition metal transporters in plants. J. Exp. Bot. 54, 2601–2613. doi: 10.1093/jxb/erg303 14585824

[B54] Hashem A. Tabassum B. Abd Allah E. F. (2019). Bacillus subtilis: A plant-growth promoting rhizobacterium that also impacts biotic stress. Saudi J. Biol. Sci. 26, 1291–1297. doi: 10.1016/j.sjbs.2019.05.004 31516360 PMC6734152

[B55] Havre G. N. (1961). The flame photometric determination of sodium, potassium and calcium in plant extracts with special reference to interference effects. Analytica Chimica Acta 25 (6), 557–6. doi: 10.1016/0003-2670(61)80134-7

[B56] Hayat K. Bundschuh J. Jan F. Menhas S. Hayat S. Haq F.. (2020). Combating soil salinity with combining saline agriculture and phytomanagement with salt-accumulating plants. Crit. Rev. Environ. Sci. Technol. 50, 1085–1115. doi: 10.1080/10643389.2019.1646087

[B57] Herliana O. Soesanto L. Mawadah E. (2018). Phytobioremediation of cadmium-contaminated soil using combination of ipomoea reptans poir and trichoderma sp. and its effect on spinach growth and yield. J. Degraded Min. Lands Manag. 6, 1519–1526. doi: 10.15243/jdmlm.2018.061.1519

[B58] Hermosa R. Viterbo A. Chet I. Monte E. (2012). Plant-beneficial effects of trichoderma and of its genes. Microbiology 158, 17–25. doi: 10.1099/mic.0.052274-0 21998166

[B59] Holmgren P. Jarvis P. G. Jarvis M. S. (1965). Resistances to carbon dioxide and water vapour transfer in leaves of different plant species. Physiol. Plant 18, 527–573. doi: 10.1111/j.1399-3054.1965.tb06917.x

[B60] Hoseinzadeh S. Shahabivand S. Aliloo A. A. (2017). Toxic metals accumulation in trichoderma asperellum and t. harzianum. Microbiology 86, 728–736. doi: 10.1134/s0026261717060066

[B61] Hui F. Liu J. Gao Q. Lou B. (2015). Piriformospora indica confers cadmium tolerance in nicotiana tabacum. J. Environ. Sci. 37, 184–191. doi: 10.1016/j.jes.2015.06.005 26574103

[B62] Ibrahim A. B. M. Zidan A. S. A. Aly A. A. M. Mosbah H. K. Mahmoud G. A.-E. (2020). Mesoporous cadmium sulfide nanoparticles derived from a new cadmium anthranilato complex: Characterization and induction of morphological abnormalities in pathogenic fungi. Appl. Organometal Chem. 34, e5391. doi: 10.1002/aoc.5391

[B63] Jagota S. K. Dani H. M. (1982). A new colorimetric technique for the estimation of vitamin c using folin phenol reagent. Anal. Biochem. 127, 178–182. doi: 10.1016/0003-2697(82)90162-2 7165085

[B64] Jia L. Liu Z. L. Chen W. He X. Y. (2012). Stimulative effect induced by low-concentration cadmium in lonicera japonica thunb. Afr J. Microbiol. Res. 6, 826–833. doi: 10.5897/AJMR11.1337

[B65] Jia L. Liu Z. Chen W. Ye Y. Yu S. He X. (2015). Hormesis effects induced by cadmium on growth and photosynthetic performance in a hyperaccumulator, lonicera japonica thunb. J. Plant Growth Regulat. 34, 13–21. doi: 10.1007/s00344-014-9433-1

[B66] Joner E. J. Briones R. Leyval C. (2000). Metal-binding capacity of arbuscular mycorrhizal mycelium. Plant Soil. 226, 227–234. doi: 10.1023/A:1026565701391

[B67] Kang S. M. Radhakrishnan R. Lee K. E. You Y. H. Ko J. H. Kim J. H.. (2015). Mechanism of plant growth promotion elicited by bacillus sp.LKE15 in oriental melon. Acta Agric. Scand. Sect. B Soil Plant Sci. 65, 637–647. doi: 10.1080/09064710.2015.1040830

[B68] Kapoor R. Bhatnagar A. K. (2007). Attenuation of cadmium toxicity in mycorrhizal celery (Apium graveolens l.). World J. Microbiol. Biotechnol. 23, 1083–1089. doi: 10.1007/s11274-006-9337-8

[B69] Khan N. Ali S. Shahid M. A. Mustafa A. Sayyed R. Z. Curá J. A. (2021). Insights into the interactions among roots, rhizosphere, and rhizobacteria for improving plant growth and tolerance to abiotic stresses: A review. Cells 10, 1551. doi: 10.3390/cells10061551 34205352 PMC8234610

[B70] Khanghahi M. Y. Pirdashti H. Rahimian H. Nematzadeh G. Sepanlou M. G. (2018). Potassium solubilising bacteria (KSB) microbed from rice paddy soil: from isolation, identification to K use efficiency. Symbiosis 76, 13e23. doi: 10.1007/s13199-017-0533-0

[B71] Khan A. R. Ullah I. Waqas M. Park G. S. Khan A. L. Hong S. J.. (2017). Host plant growth promotion and cadmium detoxification in solanum nigrum, mediated by endophytic fungi. Ecotoxicol. Environ. safety. 136, 180–188. doi: 10.1016/j.ecoenv.2016.03.014 27931714

[B72] Khan M. S. Zaidi A. Wani P. A. Oves M. (2009). Role of plant growth promoting rhizobacteria in the remediation of metal contaminated soils. Environ. Chem. Lett. 7, 1–19. doi: 10.1007/s10311-008-0155-0

[B73] Khare S. Singh N. B. Singh A. Amist N. Azim Z. Yadav R. K. (2022). Phytochemicals mitigation of brassica napus by IAA grown under cd and Pb toxicity and its impact on growth responses of anagallis arvensis. J. Biotechnol. 343, 83–95. doi: 10.1016/j.jbiotec.2021.12.001 34864124

[B74] Khyade M. S. Vaikos N. P. (2009). Phytochemical and antibacterial properties of leaves of alstonia scholaris r. Br. Afr. J. Biotechnol. 8, 6434–6436. doi: 10.5897/AJB2009.000-9489

[B75] Kim J. Rees D. C. (1994). Nitrogenase and biological nitrogen fixation. Biochemistry 33, 389–397. doi: 10.1021/bi00168a001 8286368

[B76] Koch K. (2004). Sucrose metabolism: regulatory mechanisms and pivotal roles in sugar sensing and plant development. Curr. Opin. Plant Biol. 7, 235e246. doi: 10.1016/j.pbi.2004.03.014 15134743

[B77] Kofalvi S. A. Nassuth A. (1995). Influence of wheat streak mosaic virus infection phenyl propanoid metabolism and the accumulation of phenolics and lignin in wheat. Physiol. Mol. Plant Pathol. 47, 365–377. doi: 10.1006/pmpp.1995.1065

[B78] Krizek D. T. Kramer G. F. Upadhyaya A. Mirecki R. M. (1993). UV-B response to cucumber seedlings grown under metal halide and high pressure sodium/deluxe lamps. Physiol. Plant 88, 350–358. doi: 10.1111/j.1399-3054.1993.tb05509.x

[B79] Kuan K. B. Othman R. Rahim K. A. Shamsuddin Z. H. (2016). Plant growth-promoting rhizobacteria inoculation to enhance vegetative growth, nitrogen fixation and nitrogen remobilisation of maize under greenhouse conditions. PLoS One 11, e0152478. doi: 10.1371/journal.pone.0152478 27011317 PMC4807084

[B80] Kubicek C. P. Bissett J. Druzhinina I. Kullnig-Gradinger C. Szakacs G. (2003). Genetic and metabolic diversity of trichoderma: a case study on south-East Asian isolates. Fungal Genet. Biol. 38, 310–319. doi: 10.1016/S1087-1845(02)00583-2 12684020

[B81] Kumar V. Behl R. K. Narula N. (2001). Establishment of phosphate solubilizing strains of azotobacter chroococcum in the rhizosphere and their effect on wheat cultivars under greenhouse conditions. Microbiol. Res. 156, 87–93. doi: 10.1078/0944-5013-00081 11372659

[B82] Kumar K. Khan P. (1983). Age-related changes in catalase and peroxidase activities in the excised leaves of Eleusine coracana Gaertn. cv PR 202 during senescence. Experimental Gerontology 18 (5), 409–417. doi: 10.1016/0531-5565(83)90019-0 6321215

[B83] Kumar V. Srivastava A. Jain L. Chaudhary S. Kaushal P. Soni R. (2022). Harnessing the potential of genetically improved bioinoculants for sustainable agriculture: Recent advances and perspectives. Trends Appl. Microbiol. Sustain. Economy, 319–341. doi: 10.1016/B978-0-323-91595-3.00007-0

[B84] Kumar S. Trivedi P. K. (2016). Heavy metal stress signaling in plants. Plant Metal Interaction, 585–603. doi: 10.1016/b978-0-12-803158-2.00025-4

[B85] Lamhamdi M. Bakrim A. Aarab A. Lafont R. Sayah F. (2011). Lead phytotoxicity on wheat (Triticum aestivum l.) seed germination and seedlings growth. Comptes rendus biologies. 334, 118–126. doi: 10.1016/j.crvi.2010.12.006 21333942

[B86] Lang C. A. (1958). Simple microdetermination of kjeldahl nitrogen in biological materials. Anal. Chem. 30, 1692–1694. doi: 10.1021/ac60142a038

[B87] Larcher W. (2003). Physiological plant ecology: ecophysiology and stress physiology of functional groups (Berlin, Germany: Springer-Verlag).

[B88] Li J. Chang Y. Al-Huqail A. A. Ding Z. Al-Harbi M. S. Ali E. F.. (2021). Effect of manure and compost on the phytostabilization potential of heavy metals by the halophytic plant wavy-leaved saltbush. Plants 10. doi: 10.3390/plants10102176 PMC8539195 34685988

[B89] Lichtenthaler H. K. (1987). Chlorophyll and carotenoids pigments of photosynthetic biomembranes. Methods Enzymols. 148, 350–382. doi: 10.1016/0076-6879(87)48036-1

[B90] Lim M.-S. Yeo I. W. Roh Y. Lee K.-K. Jung M. C. (2008). Arsenic reduction and precipitation by shewanella sp.: batch and column tests. Geosci. J. 12, 151–157. doi: 10.1007/s12303-008-0016-7

[B91] Lima A.d.-F. Ferreira de Moura G. Barbosa de Lima M. A. Mendes de Souza P. Alves da Silva C. A. de Campos Takaki G. M.. (2011). Role of the morphology and polyphosphate in trichoderma harzianum related to cadmium removal. Molecules 16, 2486–2500. doi: 10.3390/molecules16032486 21407149 PMC6259756

[B92] Li M. Ma G.-s. Lian H. Su X.-l. Tian Y. Huang W.-k. (2019). The effects of trichoderma on preventing cucumber fusarium wilt and regulating cucumber physiology. J. Integr. Agric. 18, 607–617. doi: 10.1016/S2095-3119(18)62057-X

[B93] Lin R. Wang X. Luo Y. Du W. Guo H. Yin D. (2007). Effects of soil cadmium on growth, oxidative stress and antioxidant system in wheat seedlings (Triticum aestivum l.). Chemosphere 69, 89–98. doi: 10.1016/j.chemosphere.2007.04.041 17568654

[B94] Liu Z. L. Chen W. He X. Y. (2012). Cadmium-induced physiological response in lonicera japonica thunb. CLEAN Soil Air Water. 41, 478–484. doi: 10.1002/clen.201200183

[B95] Liu Z. He X. Chen W. (2011). Effects of cadmium hyperaccumulation on the concentrations of four trace elements in lonicera japonica thunb. Ecotoxicology 20, 698–705. doi: 10.1007/s10646-011-0609-1 21318389

[B96] Lowry O. H. Rosebrough N. J. Farr A. L. Randall R. J. (1951). Protein measurement with the folin phenol reagent. J. Biol. Chem. 193, 291–297. doi: 10.1016/S0021-9258(19)52451-6 14907713

[B97] Lu Q. Xu Z. Xu X. Liu L. Liang L. Chen Z.. (2019). Cadmium contamination in a soil-rice system and the associated health risk: an addressing concern caused by barium mining. Ecotoxicol. Environ. safety. 183, 109590. doi: 10.1016/j.ecoenv.2019.109590 31509933

[B98] Madhava Rao K. V. Sresty T. V. (2000). Antioxidative parameters in seedlings of pigeon pea (Cajanus cajan l. millspaugh) in response to zn and Ni stresses. Plant Sci. 157, 113–128. doi: 10.1016/s0168-9452(00)00273-9 10940475

[B99] Mahmoud G. A.-E. (2021). “Microbial scavenging of heavy metals using bioremediation strategies,” in Rhizobiont in bioremediation of hazardous waste. Eds. Kumar V. Prasad R. Kumar M. (Singapore: Springer). doi: 10.1007/978-981-16-0602-1_12

[B100] Mahmoud G. A.-E. Ibrahim A. B. M. Mayer P. (2020). Zn(II) and Cd(II) thiosemicarbazones for stimulation/inhibition of kojic acid biosynthesis from aspergillus flavus and the fungal defense behavior against the metal complexes’ excesses. JBIC J. Biol. Inorganic Chem 25. doi: 10.1007/s00775-020-01802-2 32661783

[B101] Mahmoud G. A.-E. Mostafa H. H. A. (2017). Statistical optimization as a powerful tool for indole acetic acid production by fusarium oxysporum. Eur. J. Biol. Res. 7, 315–323. doi: 10.5281/zenodo.1012348

[B102] Maksymiec W. Wojcik M. Krupa Z. (2007). Variation in oxidative stress and photochemical activity in arabidopsis thaliana leaves subjected to cadmium and excess copper in the presence or absence of jasmonate and ascorbate. Chemosphere 66, 421–427. doi: 10.1016/j.chemosphere.2006.06.025 16860844

[B103] Marchel M. Kaniuczak J. Hajduk E. Właśniewski S. (2018). Response of oat (Avena sativa) to the addition cadmium to soil inoculationwith the genus trichoderma fungi. J. Elem. 23, 471–482. doi: 10.5601/jelem.2017.22.1.1391

[B104] Minguez-Mosquera M. Jaren-Galan M. Garrido-Fernandez J. (1993). Lipoxygenase activity during pepper ripening and processing of paprika. Phytochemistry 32, 1103–1108. doi: 10.1016/S0031-9422(00)95073-8

[B105] Misra H. P. Fridovich I. (1972). The role of superoxide anion in the autoxidation of epinephrine and a simple assay for superoxide dismutase. J. Biol. Chem. 247, 1972–3170. doi: 10.1016/S0021-9258(19)45228-9 4623845

[B106] Mir A. R. Alam P. Hayat S. (2022). Auxin regulates growth, photosynthetic efficiency and mitigates copper induced toxicity via modulation of nutrient status, sugar metabolism and antioxidant potential in Brassica juncea. Physiol. Biochem. 185, 244–259. doi: 10.1016/j.plaphy.2022.06.006 35717733

[B107] Mohsenzade F. Shahrokhi F. (2014). Biological removing of cadmium from contaminated media by fungal biomass of trichoderma species. J. Environ. Health Sci. Eng. 12, 102. doi: 10.1186/2052-336X-12-102 25068039 PMC4099145

[B108] Moore S. Stein W. H. (1948). Photometric ninhydrin method for use in the chromatography of amino acids. J. Biol. Chem. 176, 367–388. doi: 10.1016/S0021-9258(18)51034-6 18886175

[B109] Mukherjee S. P. Choudhuri M. A. (1983). Implications of water stress-induced changes in the levels of endogenous ascorbic acid and hydrogen peroxide in vigna seedlings. Physiologia plantarum 58, 166–170. doi: 10.1111/j.1399-3054.1983.tb04162.x

[B110] Naggar Y. A. Naiem E. Mona M. Giesy J. P. Seif A. (2014). Metals in agricultural soils and plants in Egypt. Toxicol. Environ. Chem. 96, 730–742. doi: 10.1080/02772248.2014.984496

[B111] Nahar K. Hasanuzzaman M. Alam M. M. Rahman A. Suzuki T. Fujita M. (2016). Polyamine and nitric oxide crosstalk: antagonistic effects on cadmium toxicity in mung bean plants through upregulating the metal detoxification, antioxidant defense and methylglyoxal detoxification systems. Ecotoxicol. Environ. safety. 126, 245–255. doi: 10.1016/j.ecoenv.2015.12.026 26773834

[B112] Nair A. Juwarkar A. A. Devotta S. (2008). Study of speciation of metals in an industrial sludge and evaluation of metal chelators for their removal. J. Haz. Mater. 52, 545–553. doi: 10.1016/j.jhazmat.2007.07.054 17768006

[B113] Nakano Y. Asada K. (1981). Hydrogen peroxide is scavenged by ascorbate-specific peroxidase in spinach chloroplasts. Plant Cell Physiol. 22, 867–880. doi: 10.1093/oxfordjournals.pcp.a076232

[B114] Nongmaithem N. Roy A. Bhattacharya P. M. (2016). Screening of trichoderma isolates for their potential of biosorption of nickel and cadmium. Braz. J. Microbiol. 47, 305–313. doi: 10.1016/j.bjm.2016.01.008 26991295 PMC4874587

[B115] Noor I. Sohail H. Sun J. Nawaz M. A. Li G. Hasanuzzaman M.. (2022). Heavy metal and metalloid toxicity in horticultural plants: Tolerance mechanism and remediation strategies. Chemosphere 303, 135196. doi: 10.1016/j.chemosphere.2022.135196 35659937

[B116] Ortiz A. Sansinenea E. (2022). The role of beneficial microorganisms in soil quality and plant health. Afr J. Biotechnol. 14 (9), 5358.

[B117] Pande A. Pandey P. Mehra S. Singh M. Kaushik S. (2022). Phenotypic and genotypic characterization of phosphate solubilizing bacteria and their efficiency on the growth of maize. J. Genetic Engineering Biotechnol. 15 (2), 379–391. doi: 10.1016/j.jgeb.2017.06.005 PMC6296604 30647676

[B118] Patel J. Parmar P. Dave B. Subramanian R. B. (2012). Antioxidative and physiological studies on colocasia esculentum in response to arsenic stress. Afr J. Biotechnol. 11, 16241–16246. doi: 10.5897/AJB11.3263

[B119] Patil S. V. Mohite B. V. Patil C. D. Koli S. H. Borase H. P. Patil V. S. (2020). Azotobacter. In beneficial microbes in agro-ecology: Bacteria and fungi. Elsevier. 397–426. doi: 10.1016/B978-0-12-823414-3.00019-8

[B120] Phillips K. A. Skirpan A. L. Liu X. Christensen A. Slewinski T. L. Hudson C.. (2011). Vanishing tassel encodes a grass-specific tryptophan aminotransferase required for vegetative and reproductive development in maize. Plant Cell. 23, 550–566. doi: 10.1105/tpc.110.075267 21335375 PMC3077783

[B121] Queiroz C. Mendes Lopes M. L. Fialho E. Valente-Mesquita V. L. (2008). Polyphenol oxidase: characteristics and mechanisms of browning control. Food Rev. Int. 24, 361–375. doi: 10.1080/87559120802089332

[B122] Radhakrishnan R. Lee I. J. (2016). Gibberellins producing bacillus methylotrophicus KE2 supports plant growth and enhances nutritional metabolites and food values of lettuce. Plant Physiol. Biochem. 109, 181–189. doi: 10.1016/j.plaphy.2016.09.018 27721133

[B123] Raza A. Ashraf F. Zou X. Zhang X. Tosif H. (2020). “Plant adaptation and tolerance to environmental stresses: Mechanisms and perspectives,” in Plant ecophysiology and adaptation under climate change: Mechanisms and perspectives I. Ed. Hasanuzzaman M. (Singapore: Springer). doi: 10.1007/978-981-15-2156-0_5

[B124] Rojas-Solis D. Vences-Guzmán M.Á. Sohlenkamp C. Santoyo G. (2020). Antifungal and plant growth–promoting bacillus under saline stress modify their membrane composition. J. Soil Sci. Plant Nutr. 20, 1549–1559. doi: 10.1007/s42729-020-00246-6

[B125] Sánchez-Rodríguez E. Moreno D. A. Ferreres F. Rubio-Wilhelmi M. M. Ruiz J. M. (2011). Differential responses of five cherry tomato varieties to water stress: changes on phenolic metabolites and related enzymes. Photochem 72, 723–729. doi: 10.1016/j 21420135

[B126] Schlegel H. G. (1956). Die verwertung organischer saurenduch chlorella in licht. Planta (Berl). 47, 510–526. doi: 10.1007/bf01935418

[B127] Shahabivand S. Parvaneh A. Aliloo A. A. (2017). Root endophytic fungus piriformospora indica affected growth, cadmium partitioning and chlorophyll fluorescence of sunflower under cadmium toxicity. Ecotoxicol. Environ. safety. 145, 496–502. doi: 10.1016/j.ecoenv.2017.07.064 28783599

[B128] Sheng M. Tang M. Chan H. Yang B. Zhang F. Huang Y. (2008). Influence of arbuscular mycorrhizae on photosynthesis and water status of maize plants under salt stress. Mycorrhiza 18, 287–296. doi: 10.1007/s00572-008-0180-7 18584217

[B129] Sheng X. F. Xia J. J. (2006). Improvement of rape (Brassica napus) plant growth and cadmium uptake by cadmium-resistant bacteria. Chemosphere 64, 1036–1042. doi: 10.1016/j.chemosphere.2006.01.051 16516946

[B130] Silveira J. A. G. Araújo S. A. M. Lima J. P. M. S. Viégas R. A. (2009). Roots and leaves display contrasting osmotic adjustment mechanisms in response to NaCl-salinity in atriplex nummularia. Environ. Exp. Botany. 66, 1–8. doi: 10.1016/j.envexpbot.2008.12.015

[B131] Singh G. Biswas D. R. Marwaha T. S. (2010). Mobilization of potassium from waste mica by plant growth promoting rhizobacteria and its assimilation by maize (Zea mays) and wheat (Triticum aestivum): a hydroponics study under phytotron growth chamber. J. Plant Nutr. 33, 1236–1251. doi: 10.1080/01904161003765760

[B132] Singh B. R. Steinnes E. (2020). Soil and water contamination by heavy metals. Soil Sci. (Boca Raton, Florida: CRC Press), 233–271. doi: 10.1201/9781003070184-6

[B133] Slatyer R. O. Markus D. K. (1968). Plant-water relationships. soil science Soil Sci. 106, 478. doi: 10.1097/00010694-196812000-00020

[B134] Sobkowiak R. Deckert J. (2003). Cadmium-induced changes in growth and cell cycle gene expression in suspension-culture cells of soybean. Plant Physiol. Biochem. 41, 767–772. doi: 10.1016/S0981-9428(03)00101-3

[B135] Somashekaraiah B. V. Padmaja K. Prasad A. R. K. (1992). Phytotoxicity of cadmium ions on germinating seedlings of mung bean (Phaseolus vulgaris): Involvement of lipid peroxides in chlorphyll degradation. Physiologia Plantarum. 85, 85–89. doi: 10.1111/j.1399-3054.1992.tb05267.x

[B136] Sykłowska-Baranek K. Pietrosiuk A. Naliwajski M. R. Kawiak A. Jeziorek M. Wyderska S.. (2012). Effect of l-phenylalanine on PAL activity and production of naphthoquinonepigments in suspension cultures of arnebia euchroma (Royle) johnst. In Vitro Cell Dev. Biol. Plant 48, 555–564. doi: 10.1007/s11627-012-9443-2 23049233 PMC3462983

[B137] Tatiana Z. Yamashita K. Matsumoto H. (1999). Iron deficiency induced changes in ascorbate content and enzyme activities related to ascorbate metabolism in cucumber root. Plant Cell Physiol. 40, 273–280. doi: 10.1093/oxfordjournals.pcp.a029538

[B138] USDA, FAO (2008). Oil seed situation and outlook. (Washington, DC: USDA Crop Stat., Agric. Stat.)

[B139] Van Handel E. (1968). Direct microdetermination of sucrose. Anal. Biochem. (Washington, DC: USDA Crop Stat., Agric. Stat.) 22, 280–283. doi: 10.1016/0003-2697(68)90317-5 5641848

[B140] Wani P. A. Khan M. S. (2010). Bacillus species enhance growth parameters of chickpea (Cicer arietinum l.) in chromium stressed soils. Food Chem. Toxicol. 48, 3262–3267. doi: 10.1016/j.fct.2010.08.035 20813149

[B141] Widawati S. (2018). The effect of plant growth promoting rhizobacteria (PGPR) on germination and seedling growth of sorghum bicolor l. Moench. IOP Conf. Series: Earth Environ. Sci. 166, 12022. doi: 10.1088/1755-1315/166/1/012022

[B142] Wu C. H. Wood T. K. Mulchandani A. Chen W. (2006). Engineering plant-microbe symbiosis for rhizoremediation of heavy metals. Appl. Environ. Microbiol. 72, 1129–1134. doi: 10.1128/aem.72.2.1129-1134.2006 16461658 PMC1392951

[B143] Wu F. Zhang G. Dominy P. (2003). Four barley genotypes respond differently to cadmium: lipid peroxidation and activities of antioxidant capacity. Environ. Exp. botany. 50, 67–78. doi: 10.1016/s0098-8472(02)00113-2

[B144] Yaashikaa P. R. Kumar P. S. Jeevanantham S. Saravanan R. (2022). A review on bioremediation approach for heavy metal detoxification and accumulation in plants. Environ. pollut. 301, 119035. doi: 10.1016/j.envpol.2022.119035 35196562

[B145] Yamazaki S. Ueda Y. Mukai A. Ochiai K. Matoh T. (2018). Rice phytochelatin synthases os PCS 1 and os PCS 2 make different contributions to cadmium and arsenic tolerance. Plant Direct. 2, e00034. doi: 10.1002/pld3.34 31245682 PMC6508543

[B146] Ying R. R. Qiu R. L. Tang Y. T. Hu P. J. Qiu H. Chen H. R.. (2010). Cadmium tolerance of carbon assimilation enzymes and chloroplast in Zn/Cd hyperaccumulator picris divaricata. J. Plant Physiol. 167, 81–87. doi: 10.1016/j.jplph.2009.07.005 19683362

[B147] Zafar S. Aqil F. Ahmad I. (2007). Metal tolerance and biosorption potential of filamentous fungi isolated from metal contaminated agricultural soil. Biores. Technol. 98, 2557–2561. doi: 10.1016/j.biortech.2006.09.051 17113284

[B148] Zaidi S. Usmani S. Singh B. R. Musarrat J. (2006). Significance of bacillus subtilis strain SJ 101 as a bioinoculant for concurrent plant growth promotion and nickel accumulation in brassica juncea. Chemosphere 64, 991–997. doi: 10.1016/j.chemosphere.2005.12.057 16487570

[B149] Zainab N. Amna Khan A. A. Azeem M. A. Ali B. Wang T.. (2021). PGPR-mediated plant growth attributes and metal extraction ability of sesbania sesban l. @ in industrially contaminated soils. Agronomy 11, 1820. doi: 10.3390/agronomy11091820

[B150] Zhou W. Qiu B. (2005). Effects of cadmium hyperaccumulation on physiological characteristics of sedum alfredii hance (Crassulaceae). Plant Sci. 169, 737–745. doi: 10.1016/j.plantsci.2005.05.030

[B151] Zhu T. Li L. Duan Q. Liu X. Chen M. (2020). Progress in our understanding of plant responses to the stress of heavy metal cadmium. Plant Signaling Behavior. 16, 1836884. doi: 10.1080/15592324.2020.1836884 33084518 PMC7781755

[B152] Zorzi C. Z. Garske R. P. Flôres S. H. Thys R. C. S. (2020). Sunflower protein concentrate: A possible and beneficial ingredient for gluten-free bread. Innovative Food Sci. Emerging Technologies. 66, 102539. doi: 10.1016/j.ifset.2020.102539

